# ILDR1 null mice, a model of human deafness DFNB42, show structural aberrations of tricellular tight junctions and degeneration of auditory hair cells

**DOI:** 10.1093/hmg/ddu474

**Published:** 2014-09-12

**Authors:** Eva L. Morozko, Ayako Nishio, Neil J. Ingham, Rashmi Chandra, Tracy Fitzgerald, Elisa Martelletti, Guntram Borck, Elizabeth Wilson, Gavin P. Riordan, Philine Wangemann, Andrew Forge, Karen P. Steel, Rodger A. Liddle, Thomas B. Friedman, Inna A. Belyantseva

**Affiliations:** 1 National Institute on Deafness and Other Communication Disorders, Section on HumanGenetics; 2 National Institute on Deafness and Other Communication Disorders, Molecular Biology and Genetics Section; 3 National Institute on Deafness and Other Communication Disorders, Mouse Auditory Testing Core Facility, National Institutes of Health, Bethesda, MD 20892-3729, USA; 4 Wolfson Centre for Age-Related Diseases, King's College London, London SE1 1UL, UK; 5 Department of Medicine, Duke University Medical Center, Durham, NC 27710, USA; 6 Institute of Human Genetics, University of Ulm, Ulm 89081, Germany; 7 Anatomy and Physiology Department, Kansas State University, Manhattan, KS 66506-5802, USA and; 8 Centre for Auditory Research, University College London, London WC1X 8EE, UK

## Abstract

In the mammalian inner ear, bicellular and tricellular tight junctions (tTJs) seal the paracellular space between epithelial cells. Tricellulin and immunoglobulin-like (Ig-like) domain containing receptor 1 (ILDR1, also referred to as angulin-2) localize to tTJs of the sensory and non-sensory epithelia in the organ of Corti and vestibular end organs. Recessive mutations of *TRIC (DFNB49)* encoding tricellulin and *ILDR1* (*DFNB42*) cause human nonsyndromic deafness. However, the pathophysiology of DFNB42 deafness remains unknown. ILDR1 was recently reported to be a lipoprotein receptor mediating the secretion of the fat-stimulated cholecystokinin (CCK) hormone in the small intestine, while ILDR1 in EpH4 mouse mammary epithelial cells *in vitro* was shown to recruit tricellulin to tTJs. Here we show that two different mouse *Ildr1* mutant alleles have early-onset severe deafness associated with a rapid degeneration of cochlear hair cells (HCs) but have a normal endocochlear potential. ILDR1 is not required for recruitment of tricellulin to tTJs in the cochlea *in vivo*; however, tricellulin becomes mislocalized in the inner ear sensory epithelia of ILDR1 null mice after the first postnatal week. As revealed by freeze-fracture electron microscopy, ILDR1 contributes to the ultrastructure of inner ear tTJs. Taken together, our data provide insight into the pathophysiology of human DFNB42 deafness and demonstrate that ILDR1 is crucial for normal hearing by maintaining the structural and functional integrity of tTJs, which are critical for the survival of auditory neurosensory HCs.

## INTRODUCTION

A variety of intercellular structures seal the paracellular space between epithelial cells that outline and separate different cavities and structures in the body. Hemidesmosomes, desmosomes and adherens junctions compose adhesion junctions between neighboring epithelial cells while gap junctions provide an intercellular communication pathway (1). Tight junctions (TJs) between adjacent epithelial cells are ion- and size-selective barriers that regulate the lateral diffusion of solutes and water molecules through the paracellular pathways (2,3). Bicellular tight junctions (bTJs) form a network of parallel, paired strands comprised of integral membrane proteins, membrane-associated and intracellular proteins (3,4). These protein complexes of paired TJ strands from two adjacent cells associate laterally with one another to contribute to the semi-permeable seals formed within the paracellular space. On the intracellular side of the plasma membrane, a variety of adaptor proteins are involved in the formation of TJs, such as zona occludens (ZO)-1, ZO-2 and ZO-3, which connect TJs with the actin cytoskeleton, signaling molecules as well as membrane proteins (3,5).

Many integral membrane TJ proteins have been identified, including a family of 24 different claudins (2), junctional adhesion molecules and the TJ-associated MAL and related proteins for vesicle trafficking and membrane link (MARVEL) proteins occludin (6), tricellulin (also known as marvelD2) (7,8) and marvelD3 (3,9). Occludin was the first component of TJs to be identified (6) and has been shown to form heteromeric complexes with tricellulin (10). Claudins play a key role in TJ formation through lateral association and homotypic adhesive interactions between claudins in adjacent cells (3). Claudin-based pores formed between cells in different epithelia show variation in permeability and ionic charge preferences, thus creating a wide range of functionally distinct TJ paracellular barriers throughout the body (2).

**Figure 1.**
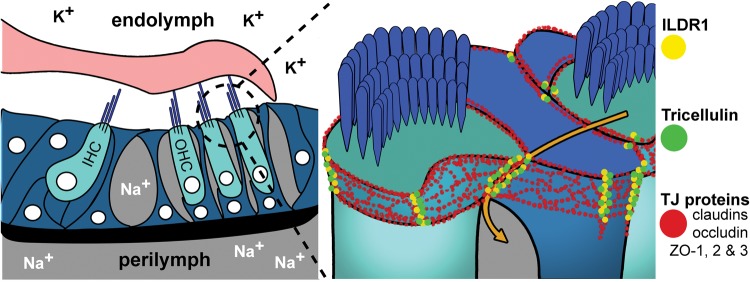
Tight junction composition and function in the mammalian inner ear. The bTJs and tTJs are composed of integral membrane and membrane-associated proteins that form paired, parallel horizontal and vertical strands with those in adjacent cells (red). In the inner ear, bTJs and tTJs help separate K^+^ rich endolymph from Na^+^ rich perilymph. Tricellulin (green) and ILDR1 (yellow) localize to tricellular contacts of both sensory and non-sensory epithelia. In the area where three cells meet, tTJ components form a central tube, which is responsible for most macromolecule paracellular flux (orange arrow).

In the mammalian cochlea, TJs separate the endolymphatic, perilymphatic and intrastrial fluid spaces, three compartments with distinctly different fluid compositions (11–13). Additionally, TJs play a key role in the generation and maintenance of the endocochlear potential (EP) of 80–120 mV, essential for normal hearing, by sealing epithelial paracellular spaces to separate the potassium-rich endolymph from sodium-rich perilymph (Fig. 1) (14). The bTJs of the reticular lamina have a unique, elaborate structure (11) composed of a fusion of TJs and tight adherens junctions (TAJs) (15). These TAJs are large and, as revealed by freeze-fracture, characterized by strands present along the entire junction depth unlike TJs in most epithelial cells (11).

Mutations of several genes encoding TJ proteins have been found to cause deafness in humans, mice and zebrafish (Table 1). Mutant alleles of human *CLDN14*, encoding claudin 14, cause nonsyndromic deafness DFNB29 (20). Deaf, claudin-11-deficient mice lack an EP due to impaired TJs between basal cells of the stria vascularis (23,24). In contrast, mutations in *Cldn9*, encoding claudin 9, and *Cldn14* have altered TJs of the reticular lamina but are able to maintain a normal EP (21,22). Hearing loss in both of these mouse models was attributed to leakage of potassium ions through the paracellular pathway of the organ of Corti reticular lamina into the basolateral domain. The increased concentration of potassium in the perilymph surrounding free lateral surfaces of outer hair cells (OHCs) creates a toxic microenvironment, contributing to progressive hair cell (HC) loss in both of these mouse models during the late postnatal period when the EP and hearing are being established in the mouse (21,22).

**Table 1.** Hearing loss as an autosomal recessive phenotype associated with mutations^a^ of genes encoding TJ proteins

Species	Gene	Auditory phenotype	Reference
Human	*ILDR1*	Moderate-to-profound DFNB42, prelingual deafness. OMIM: 609646	(16–18)
Human	*TRIC*	Moderate-to-profound DFNB49 deafness. OMIM: 610153	(8)
Mouse	*Tric*	Profound DFNB49 deafness and loss of sensory HCs. OMIM: 610153	(19)
Human	*CLDN14*	Profound, congenital, DFNB29 deafness. OMIM: 614035	(20)
Mouse	*Cldn14*	Profound deafness and loss of HCs. OMIM: 614035	(21)
Mouse	*Cldn9*	Profound deafness and loss of HCs	(22)
Mouse	*Cldn11*	Profound deafness and low EP	(23,24)
Zebrafish	*cldnj*	Impaired hearing and vestibular function	(25)

OMIM, Online Mendelian Inheritance in Man, http://www.omim.org/.

a No dominant mutant alleles encoding TJ proteins have been reported to date.

Tricellular tight junctions (tTJs) serve as sealing elements of the central tube formed when the parallel-paired strands of TJs from three adjacent cells come together and extend basally at the location where three epithelial cells meet (4,26–28). While bTJs are responsible for approximately 99% of the paracellular ion transport, tTJs allow for the passage of various molecules and macromolecules through the tricellular central tube with tricellulin expression regulating the size and selectivity of the pore (26). Tricellulin was the first identified component of tTJs and mutations of *TRIC*, encoding tricellulin, are associated with human nonsyndromic recessive deafness DFNB49 (7,8).

Immunoglobulin-like domain-containing receptor 1 (ILDR1, also referred to as angulin-2) was recently reported as a lipoprotein receptor mediating the secretion of fat-stimulated cholecystokinin (CCK) hormone in the small intestine (29) and was also identified as a tTJ protein (30). Mutations of human *ILDR1* cause nonsyndromic deafness DFNB42 (16). In the mouse organ of Corti, ILDR1 localizes to tTJ of both sensory and non-sensory epithelia (30). ILDR1 is a member of the angulin family of tTJ-associated proteins that includes angulin-1/lipolysis-stimulated lipoprotein receptor (LSR), angulin-2/ILDR1 and angulin-3/ILDR2 (30,31). Angulin proteins were reported to recruit tricellulin to the tTJs in cultured EpH4 mammary epithelial cells suggesting a dependence of tricellulin on angulin proteins for proper tTJ localization during development (30).

**Figure 2.**
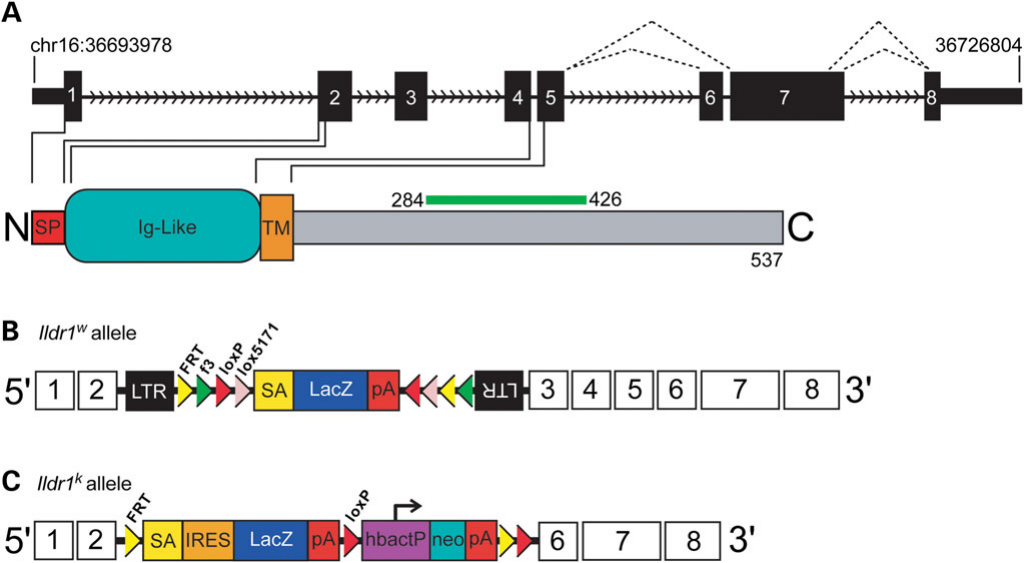
Schematic representation of ILDR1 and the two mutant alleles of mouse *Ildr1* used in this study. (**A**) ILDR1 is a 537 amino acid protein encoded by 8 exons and has an N-terminal signal peptide (red), an Ig-like domain (teal) and a transmembrane domain (orange). To date, three isoforms of ILDR1 have been reported. Exon 6 is alternatively spliced and exon 7 has two donor splice sites (black dotted lines).The epitope for the commercial ILDR1 antibody (Sigma-Aldrich) used in this study, encoded by a portion of exon 7, is shown as green bar above the protein. (**B**) Gene trap allele of *Ildr1^Gt(D178D03)Wrst^* (*Ildr1^w−/−^*) was produced from insertion of targeting vector (rFlipROSAβgeo (Cre)) into intron 2 of *Ildr1* (197 bp internal to the exon 2/intron 2 boundary). (**C**) *Ildr1^tm1(KOMP)Wtsi^* allele (*Ildr1^k−/−^*) was generated by targeted replacement of exons 3–5 with a Lac-Z cassette.

In this study, the effect of loss of ILDR1 (Fig. 2A) on hearing function was characterized using two different functional null mutant alleles of mouse *Ildr1:Ildr1^Gt(D178D03)Wrst^*, a gene trap in intron 2, henceforth abbreviated *Ildr1^w−/−^* (Fig. 2B) and *Ildr1^tm1(KOMP)Wtsi^*, which deletes exons 3–5 and designated *Ildr1^k−/−^* (Fig. 2C). As a genomic manipulation can have unexpected off-target effects, the link between a gene and a phenotype is strengthened if the same key features of the phenotype are observed for more than one engineered mutant allele. The hearing phenotype of *Ildr1^w−/−^* and *Ildr1^k−/−^* mice, which are on different genetic backgrounds, was assessed by auditory brainstem response (ABR), distortion product otoacoustic emission (DPOAE) and EP measurements. Furthermore, the inner ear phenotype of *Ildr1^w−/−^* mice was studied by immunocytochemistry, scanning electron microscopy (SEM), and freeze-fracture electron microscopy.

Whether or not ILDR1 is necessary to recruit tricellulin to tTJs *in vivo*, specifically in the inner ear, is an unanswered question. The mechanism that targets tricellulin to the tTJs in the cochlea is not well understood and is addressed in our study. Our results indicate that ILDR1 is not necessary for tricellulin recruitment to tTJs in the auditory and vestibular sensory epithelia or in the stria vascularis, but it is essential for retention of tricellulin and the long-term integrity of tTJs. Additionally, we provide insight into potential mechanisms for deafness due to the loss of ILDR1 function.

## RESULTS

### In wild-type mice, ILDR1 is localized to tTJs of the inner ear

**Figure 3.**
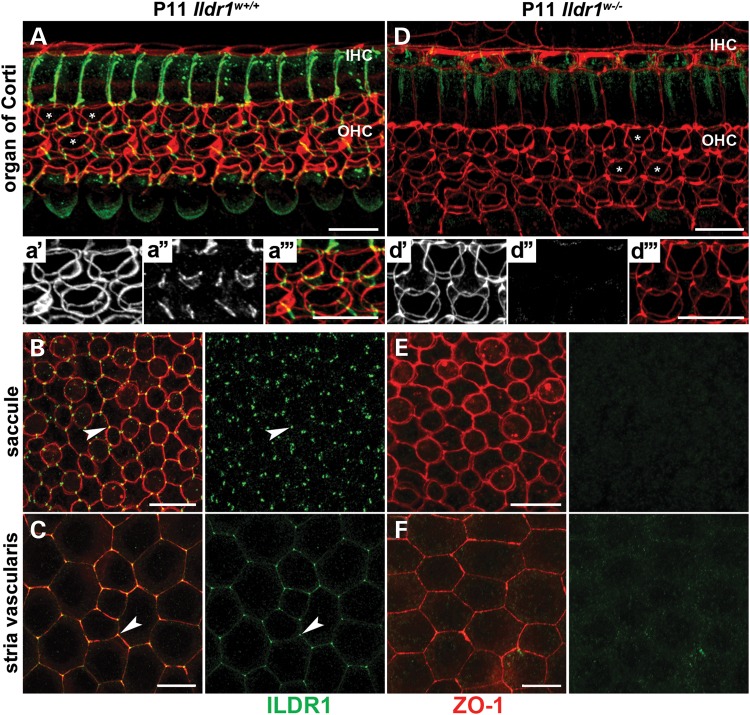
ILDR1 localizes to tTJs between epithelial cells of the inner ear and is absent in *Ildr1^w−/−^* mice. In wild-type mice, (**A**) 3D reconstruction image from a confocal z-stack shows ILDR1 (green) localization along the entire depth of the tTJ between HCs and supporting cells, connecting the circumferential rings of ZO-1 (red) at the top and bottom of these junctions (a’–a’’’**)**. Localization of ILDR1 at tTJs between (**B**) HCs and supporting cells of vestibular sensory epithelia and (**C**) between stria vascularis marginal cells of *Ildr1^w+/+^* mice. No ILDR1 was detected in *Ildr1^w−/−^* mice (**D–F**). Asterisks in (A) and (D) indicate magnified HCs. Arrowheads in (B) and (C) point to ILDR1 localization at tricellular contacts. Scale bars: 10 µm.

ILDR1 immunoreactivity spans the entire depth of the tTJ as seen in the control *Ildr1^w^**^+/+^***organ of Corti labeled with anti-ILDR1 and anti-ZO-1 antibodies (Fig. 3A). TJ-associated protein ZO-1 outlines the OHC cuticular plate in two rings connected by spokes. An upper heart-shape ring defines the apical surface of OHCs, while a lower circular ring corresponds to the shape of the bottom portion of the OHC cuticular plate (Fig. 3A). In wild-type mice, ILDR1 highlights the central tube of tricellular junctions formed by the lateral membranes of the three adjacent cells and is observed along the entire depth of HC tTJs (Fig. 3A). Heterozygous (*Ildr1^w+/−^*) mice showed staining indistinguishable from *Ildr1^w+/+^* mice (data not shown). Thus, in the wild-type mouse inner ear, ILDR1 is present along the entire depth of the organ of Corti tTJs demarcated by two rings of ZO-1 that flank the upper and lower border of the tTJ. ILDR1 was also detected in tTJs of the vestibular sensory epithelia (Fig. 3B) and in marginal cells of the stria vascularis (Fig. 3C), as well as in other epithelial cells of the inner ear of *Ildr1^w^**^+/+^*** mice (data not shown).

### *Ildr1^w−/−^* mice lack ILDR1 at tTJs in the inner ear

The *Ildr1^w−/−^* allele was developed previously to investigate the function of ILDR1 in the small intestine (29). Since the inner ear of *Ildr1^w−/−^* mice had not previously been examined for ILDR1 localization, we used anti-ILDR1 antibodies to study its localizations in the inner ear sensory epithelium. These mice were also used to examine the specificity of the antiserum against ILDR1. We found that *Ildr1^w−/−^* mice have no detectable ILDR1 in tTJs of cochlear and vestibular (saccule, ampules and utricle) sensory epithelia, marginal cells of stria vascularis (Fig. 3D–F) and other epithelial cells of the inner ear (data not shown).

### *Ildr1^w−/−^* and *Ildr1^k−/−^* mice are deaf

**Figure 4.**
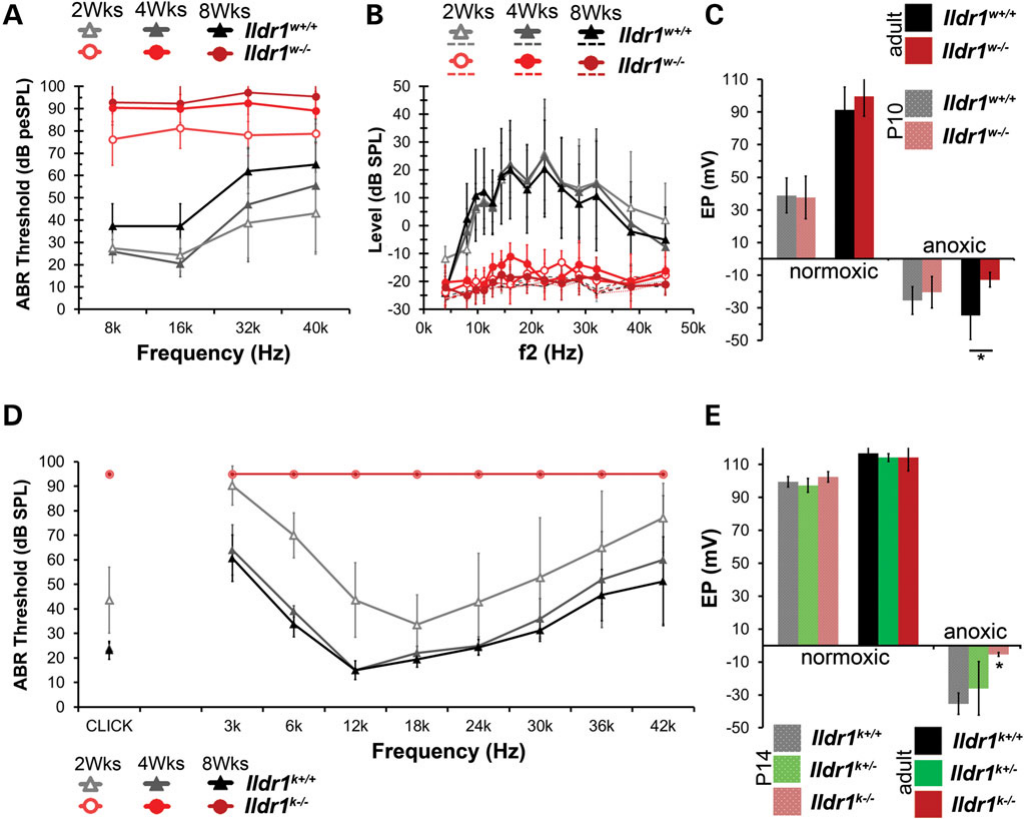
*Ildr1^w−/^*^−^ mice are severely hearing impaired but have a normal EP. (**A**) Mean ABR thresholds of *Ildr1^w^*^−/−^ and *Ildr1^w+/+^* littermates at 2 weeks (*n* = 8 each), 4 weeks (*n* = 11 each) and 8 weeks of age (*n* = 11 each). *Ildr1^w^*^−/−^ mice have higher ABR thresholds than those of *Ildr1^w+/+^* littermates starting at 2 weeks, which increased into adulthood. *Ildr1^w+/−^* mice have a hearing phenotype indistinguishable from *Ildr1^w+/+^* littermates (Supplementary Material, Fig. S1). (**B**) Mean DPOAE levels for the same *Ildr1^w−/−^* and *Ildr1^w+/+^* littermates tested with ABRs. *Ildr1^w−/−^* mice have no detectable DPOAE levels at 2, 4 and 8 weeks of age (*n* = 8, 11, and 11, respectively, per genotype). Dotted lines represent noise floor corresponding to each age and genotype. DPOAEs of *Ildr1^w+/−^* mice were indistinguishable from *Ildr1^w+/+^* littermates at all three ages tested (Supplementary Material, Fig. S1). (**C**) EP measurements from adult (P56-85) and P10 *Ildr1^w−/−^* and *Ildr1^w+/+^* littermates. P10 *Ildr1^w−/−^* mice establish normal EPs under both normoxic and anoxic conditions (*n* = 6 and 7 for *Ildr1^w+/+^* and *Ildr1^w−/−^*, respectively). However, the negative EP under anoxic conditions was smaller in adult *Ildr1^w−/−^* mice compared with *Ildr1^w+/+^* mice (Student's *t*-test, *P* < 0.05, *n* = 5, *Ildr1^w+/+^*, *n* = 4, *Ildr1^w−/−^*) while the positive EP in adult *Ildr1^w−/−^* mice developed a normal magnitude (*n* = 7, *Ildr1^w+/+^*and *Ildr1^w−/−^*). (**D**) Mean ABR thresholds of *Ildr1^k^*^−/−^ and *Ildr1^k+/+^* littermates at 2 weeks (*n* = 7 each), 4 weeks (*n* = 5 *Ildr1^k+/+^,* 6 *Ildr1^k^*^−/−^) and 8 weeks of age (*n* = 8 *Ildr1^k+/+^,* 12 *Ildr1^k^*^−/−^). *Ildr1^k^*^−/−^ mice showed no responses at 95 dB SPL, the maximum sound level used. Difference in ABR threshold elevation between *Ildr1^w−/−^* and *Ildr1^k−/−^* mice by the second postnatal week may due to variation in the genetic background. *Ildr1^w−/−^* and *Ildr1^k−/−^* are on Swiss Webster and C57BL/6N genetic backgrounds, respectively. Mutant alleles affecting hearing on a C57BL/6 background have a more severe deafness phenotype (32). (**E**) Positive EP and negative EP measurements made in P14 *Ildr1^k+/+^*, *Ildr1^k+/−^* and *Ildr1^k^*^−/−^ mice. No significant difference was found for positive EP measurements (ANOVA, *P* > 0.05). However, the anoxic EP showed a significantly lower magnitude in the *Ildr1^k^*^−/−^ compared with the *Ildr1^k+/+^* and *Ildr1^k+/−^* controls (ANOVA, *P* < 0.01). Positive EP measurements made in 10 week old *Ildr1^k+/+^*, *Ildr1^k+/−^* and *Ildr1^k^*^−/−^ mice showed no significant difference (ANOVA, *P* > 0.05). Error bars (A–E) are shown as ±SD.

To investigate the effect of ILDR1 loss on hearing in *Ildr1^w−/−^* mice, we measured ABRs at four frequencies (8, 16, 32 and 40 kHz) (Fig. 4A). *Ildr1^w−/−^* mice have severe hearing loss by the second postnatal week, shortly after the onset of hearing in wild-type mice, compared with both *Ildr1^w+/+^* and *Ildr1^w+/−^* littermates. By 8 weeks of age, the hearing loss is profound in homozygous mutant mice (Fig. 4A). In most cases, no measurable ABR responses were detected at the maximum level tested (90 dB peSPL). *Ildr1^w+/+^* and *Ildr1^w+/−^* littermates show elevated thresholds at higher frequencies in an age-dependent manner, which likely is attributed to the background strain (Swiss Webster, Charles River Laboratories International Inc.) of these mice (33). However, thresholds from *Ildr1^w+/+^* and *Ildr1^w+/−^* littermates were still markedly lower than that of *Ildr1^w−/−^* mice (Supplementary Material, Fig. S1A). These results indicate that hearing loss in *Ildr1^w−/−^* mice is an early-onset recessive trait that is rapidly progressive, and severe.

Responses to tone pips (3–42 kHz) and click stimuli were measured in *Ildr1^k−/−^* mice of each genotype aged 2, 4 and 8 weeks (Fig. 4D). No ABRs were detected for any of the test stimuli for *Ildr1^k−/−^* mice at the ages tested (Fig. 4D). For these mice, threshold was arbitrarily assigned to be 95 dB SPL. At each age, the mean thresholds for *Ildr1^k+/−^* mice were comparable to that of *Ildr1^k+/+^* mice across all frequencies (Supplementary Material, Fig. S1C). Therefore, hearing loss in the *Ildr1^k−/−^* mouse is also profound and an early-onset recessive trait.

To evaluate the functional activity of OHCs in *Ildr1^w−/−^* mice, we measured DPOAEs (Fig. 4B). Both wild-type and heterozygous littermate controls had measurable DPOAEs at 2, 4 and 8 weeks of age (Supplementary Material, Fig. S1B). However, DPOAEs were absent for *Ildr1^w−/−^* littermates by the second postnatal week, and *Ildr1^w−/−^* mice did not have measurable responses at 4 and 8 weeks of age compared with *Ildr1^w+/+^* littermates (Fig. 4B). These data indicate that in the *Ildr1^w−/−^* mouse there is an early postnatal loss of OHC function.

### *Ildr1^w−/−^*and *Ildr1^k−/−^* mice have a normal EP

Alterations in TJ barriers of the inner ear may affect the EP. The EP is the transepithelial voltage across the epithelial barrier in the cochlea. A positive EP with a magnitude of 80–100 mV, variation dependent on strain or species, is required for normal hearing. The positive EP is generated by the stria vascularis and depends on intact epithelial barriers, normoxic metabolism and normal ionic conductances in the stria vascularis (13). Furthermore, under acute anoxic conditions, cochlear epithelia generate a negative EP with a magnitude of 30–40 mV. The negative EP depends on intact epithelial barriers and normal ionic conductances, primarily the apical transduction channel and the basolateral potassium channels in OHCs (34). Thus, measurements of the positive and negative EP can reveal a variety of defects in the cochlea. We first evaluated the positive EP under normoxic conditions when an anesthetized animal is maintained under normal tissue oxygen levels by spontaneous breathing and the negative EP under anoxic conditions when tissue oxygen levels were depleted due to cessation of spontaneous breathing (Fig. 4C and E). Evaluations were performed in young (P10) and adult (P56-85) *Ildr1^w−/−^* mice and *Ildr1^w+/+^* littermates (Fig. 4C). To investigate if the loss of ILDR1 affects the development of the EP, we tested *Ildr1^w^* mice around the time the EP is established (P10). At this age, there was no significant difference in either the positive EP between *Ildr1^w+/+^* (39 ± 11 mV, *n* = 6) and *Ildr1^w−/−^* (38 ± 13 mV, *n* = 7) mice or the negative EP (−26 ± 9 mV, *n* = 6–20 ± 10 mV, *n* = 7 for *Ildr1^w+/+^* and *Ildr1^w−/−^*, respectively, Fig. 4C), indicating that at this age the epithelial barriers of the inner ear, including the organ of Corti and stria vascularis as well as apical and basolateral conductances of the OHCs, were apparently intact. Furthermore, adult *Ildr1^w−/−^* mice had a positive EP of normal magnitude (100 ± 12 mV, *n* = 7) compared with *Ildr1^w+/+^* littermates (91 ± 14 mV, *n* = 7, Fig. 4C), indicating that the barrier function of the cochlear epithelia was not impaired and that stria vascularis was still functional. However, the negative EP in adult *Ildr1^w−/−^*mice was significantly smaller (−13 ± 5 mV, *n* = 4, Student's *t*-test, *P* < 0.05) when compared with *Ildr1^w+/+^* mice (−35 ± 15 mV, *n* = 5) (Fig. 4C) (35,36). These data indicate that lack of ILDR1 does not cause a delay in establishing the EP or a compromise of the stria vascularis in maintaining the positive EP. Diminution of the negative EP, however, could be the consequence of degeneration of the organ of Corti that results from the lack of ILDR1.

EP was also recorded in *Ildr1^k^* mice at P14 and 10 weeks of age (Fig. 4E). At P14, there were no significant differences (analysis of variance (ANOVA), *P* > 0.05) in the magnitude of the positive EP between *Ildr1^k+/+^* (100 ± 3 mV, *n* = 4), *Ildr1^k+/−^* (97 ± 4 mV, *n* = 6) or *Ildr1^k−/−^* (102 ± 3 mV, *n* = 4) mice (Fig. 4E). Similarly, the magnitude of the positive EP in 10 week old mice was not significantly altered (ANOVA, *P* > 0.05) in *Ildr1^k+/+^* (117 ± 6 mV, *n* = 4), *Ildr1^k+/−^* (114 ± 2 mV, *n* = 4) or *Ildr1^k−/−^* mice (114 ± 8 mV, *n* = 7). However, the negative EP recorded under anoxic conditions in P14 *Ildr1^k−/−^* mice (−5 ± 1 mV, *n* = 4) was significantly smaller (ANOVA, *P* < 0.01) than that recorded in *Ildr1^k+/+^* (−35 ± 7 mV, *n* = 4) and *Ildr1^k+/−^* (−26 ± 16 mV, *n* = 6) mice (Fig. 4E). Taken together, the normal magnitude of the positive EP in *Ildr1^k−/−^* mice strengthens the conclusion that ILDR1 is neither essential for establishing nor for maintaining a normal positive EP.

### ILDR1-deficient mice exhibit rapid base-to-apex OHC degeneration

**Figure 5.**
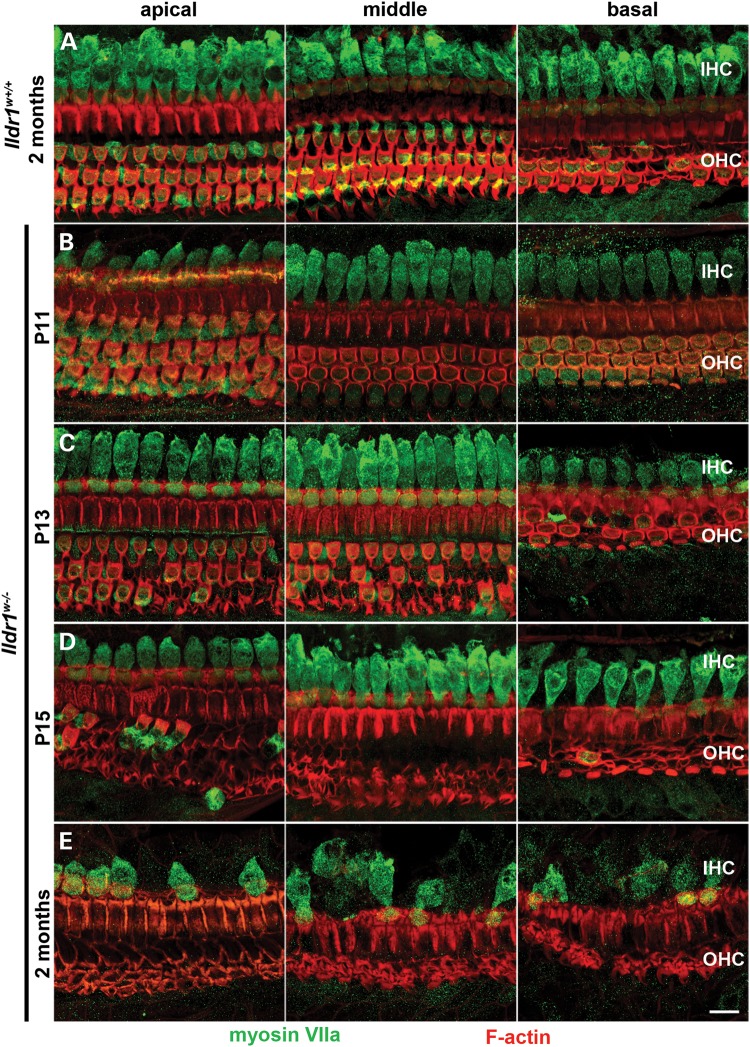
OHCs of *Ildr1^w^*^−/−^ mice rapidly degenerate around hearing onset followed by a slow IHC loss in adult mice. Maximum intensity projections show degeneration of auditory HCs in *Ildr1^w^*^−/−^ mice. Hair cell bodies were probed with antibody against myosin VIIa (green) and F-actin was labeled with phalloidin (red). (**A**) Apical, middle and basal sensory epithelium in adult, 2 month old *Ildr1^w^*^+/+^ control mice. (**B–E**) Apical, middle and basal sensory epithelium from P11 *Ildr1^w−^*^/−^, P13 *Ildr1^w^*^−/−^, P15 *Ildr1^w^*^−/−^, and adult *Ildr1^w^*^−/−^ mice indicate that HCs develop normally but OHCs begin to degenerate around hearing onset. (E) In adult, 2 month old *Ildr1^w−/−^* mice, gradual loss of IHCs is observed. Scale bar: 10 µm.

To investigate the inner ear morphology of ILDR1 null mice, we immunostained the sensory epithelium using myosin VIIa antiserum as a HC marker (37). In control wild-type mice at 2 months of age, there is no OHC loss in the apical and middle turns of the organ of Corti (Fig. 5A), although some loss of OHCs is detected in the basal turn, which likely reflects the Swiss Webster genetic background (Fig. 5A, right panel). In *Ildr1^w−/−^* mice, no loss of OHCs is observed at P11 (Fig. 5B). However, degeneration of OHCs is detected at P12 (data not shown) and becomes prominent at P13-P15 (Fig. 5C and D). OHC demise occurs in a mosaic pattern and progresses rapidly until there is a complete loss of OHCs by 1 month of age (Supplementary Material, Fig. S2A–C). *Ildr1^w+/+^* and *Ildr1^w+/−^* mice did not show signs of OHC degeneration at this time (Supplementary Material, Fig. S2A and B).

Compared with OHCs, inner hair cell (IHC) degeneration progressed more slowly with pronounced cell death at 2 months of age (Fig. 5B–E). The early-onset and severe hearing loss in the *Ildr1^w−/−^* mouse coincides with the degeneration of OHCs (37). Similarly, by P14, *Ildr1^k−/−^* OHCs are degenerating with some OHCs remaining in the apical 20% of the cochlear duct (data not shown). In contrast, *Ildr1^w−/−^* vestibular HCs at 2 months of age are intact as revealed by confocal microscopy (Supplementary Material, Fig. S2D and E). Consistent with these observations, we did not notice any obvious vestibular dysfunction in affected DFNB42 individuals (16) and no head tossing or circling in *Ildr1^w−/−^* mice, although vestibular evoked potentials (VsEPs) were not tested in these mice (38).

### Abnormalities of stereocilia in *Ildr1^w−/−^* mice portend OHC loss

**Figure 6.**
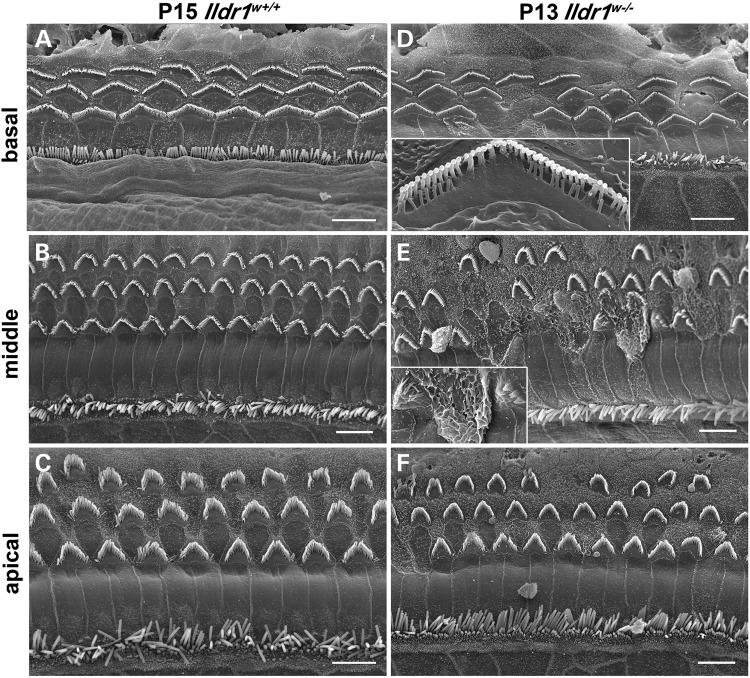
SEM images show OHC degeneration in the organ of Corti from P13 *Ildr1^w−/−^* mice in comparison to P15 *Ildr1^w^*^+/+^ controls. (**A**, **D**) basal turn, (**B**, **E**) middle turn and (**C**, **F**) apical turn. Note degeneration and loss of OHCs in all three turns of *Ildr1^w−/−^* mice. Inset in (D) shows a close-up view of a degenerating OHC from another image of the basal turn. Note the loss of shorter-row stereocilia. Inset in (E) shows close-up of degenerating OHC apical membrane fusion. IHC degeneration is not evident at P13. Scale bars: 5 mm.

Using SEM analysis, we show the normal appearance of apices of IHCs and OHCs of control P15 *Ildr1^w+/+^* mice (Fig. 6A–C). In P13 *Ildr1^w−/−^* mouse organ of Corti, IHC stereocilia bundles also do not have any obvious abnormalities, while OHCs show a mosaic pattern of degeneration and loss. We observed more pronounced loss of OHCs at the basal and middle turns of the cochlea of P13 *Ildr1^w−/−^* mouse (*n* = 4) compared with the apical turn (Fig. 6D and F), which is consistent with the immunocytochemistry data (Fig. 5C and D). Moreover, in P13 *Ildr1^w−/−^* mice, degenerating OHC stereocilia bundles exhibited partial loss of shorter-row stereocilia and fusion of stereocilia with the apical membrane of HCs (Fig. 6D, E, insets). Several HCs had apical membrane distortions and missing stereocilia bundles (Fig. 6E). Subsequently, areas lacking OHCs in the middle turn of the organ of Corti appeared to be populated only with supporting cells that often have enlarged or misshapen apical surfaces occupying the vacant OHC space thus sealing the reticular lamina (Fig. 6E). These changes to the hair bundles at P13 may be a consequence of prior damage elsewhere in the cell body, with subsequent apoptotic loss of OHCs as the cause of rapid progression to profound hearing loss. Because there is a normal negative EP in P10 *Ildr1^w−/−^* mice indicating normal OHC function, we investigated if OHC loss or stereocilia abnormalities could be detected by P11. No obvious stereocilia abnormalities or OHC loss was detected by SEM at P11 in the *Ildr1^w−/−^* mouse organ of Corti (Supplementary Material, Fig S3) confirming the myosin VIIa immunostaining results at P11 (Fig. 5B). Our SEM data suggest the importance of ILDR1 for OHC survival in the organ of Corti immediately after the onset of hearing in mouse.

### Tricellulin phenotype at tTJs in the absence of ILDR1 in the inner ear of *Ildr1^w−/−^* mice

**Figure 7.**
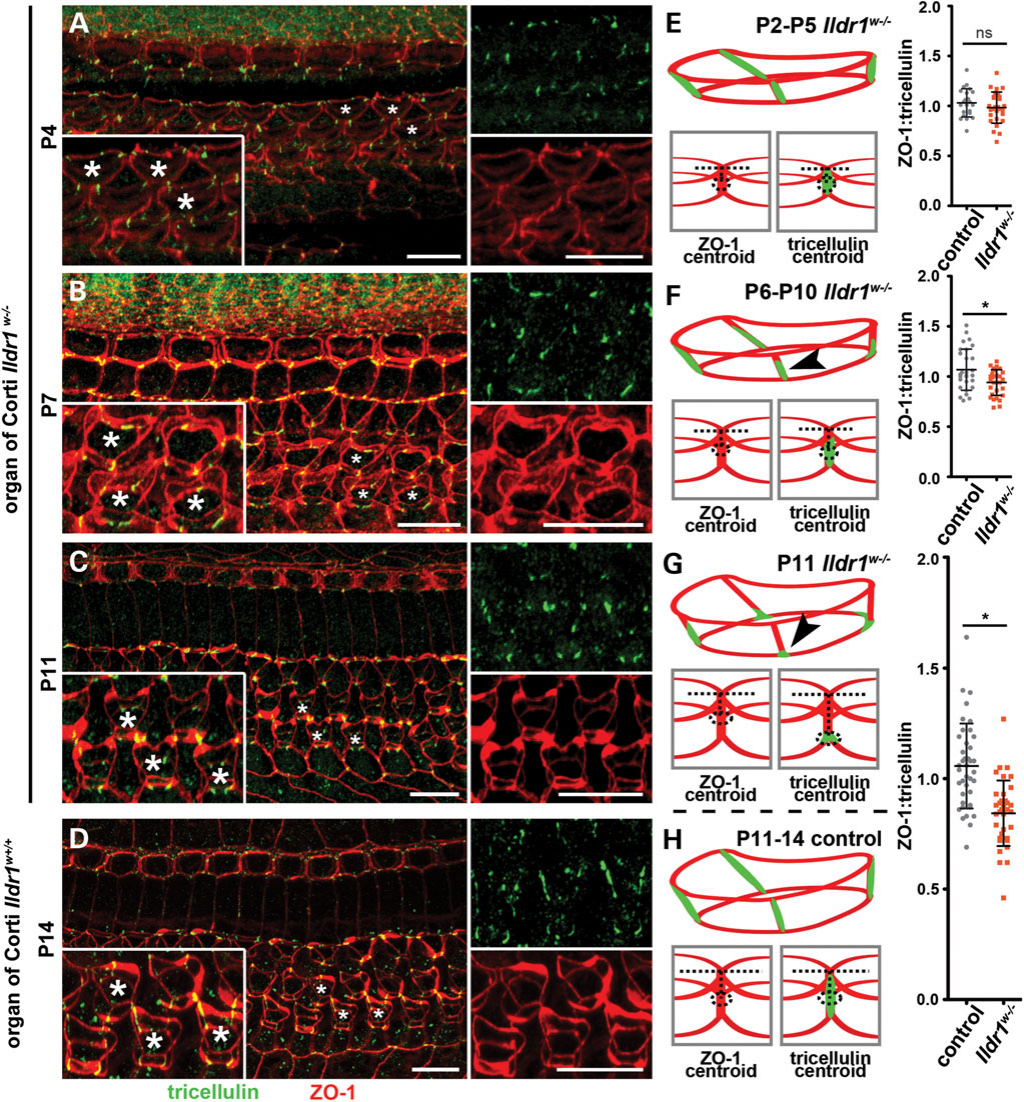
Tricellulin localizes to tTJs in the absence of ILDR1, prior to degeneration of auditory HCs. (**A–D**) 3D reconstructions of z-stack confocal images from whole-mount organ of Corti samples, probed with antibodies directed against tricellulin (green) and ZO-1 (red). (A) In P4 *Ildr1^w−/−^* mice, tricellulin is visualized along the entire tTJ depth. (**E)** Cartoon schematic of tricellulin localization. Velocity measurement parameters and graph show that tricellulin is localized normally in P2–P5 *Ildr1*^w−/−^ mice (mean age = 3.81 ± 1.19) compared with control animals, *P* > 0.05, *n* = 4 control animals and 5 experimental animals. (B) By P7 (mean age of P6–P10 group = 7.54 ± 0.72 SD), some mislocalization begins to be observed in *Ildr1^w−/−^* mice. (**F**) Arrowhead in cartoon depicts mislocalization at P7, *P* < 0.005, *n* = 5 animals per group. (C) In P11 *Ildr1^w−/−^* mice, mislocalization is apparent due to lack of tricellulin signal toward the top of the tTJ and its concentration near the basal end of the tTJ. (**G**) Arrowhead in cartoon shows interpreted mislocalization at P11; and velocity measurements and graph for the P11–P14 control group show a clear statistical significant mislocalization of tricellulin, *P* < 0.000001, *n* = 6 animals per group. 3D reconstruction of (**D–H**) *Ildr1^w^*^+/+^ control mice show tricellulin localization as outlined in the cartoon. Drawings and arrowheads show interpretation of tricellulin mislocalization in *Ildr1^w−/−^* mice as the cuticular plate thickens from P4 to P11 and visual description of Velocity measurements. Insets show high magnification of OHCs from the corresponding age and asterisks indicate HCs depicted in magnified panels. Error bars (E–H) are shown as ±SD. Scale bars 10 µm.

To investigate if the absence of ILDR1 affects recruitment or retention of tricellulin *in vivo*, we investigated tricellulin localization in the *Ildr1^w−/−^* inner ear. Tricellulin has been shown to localize to tTJs of all the epithelial cells that outline the scala media including the tTJs of HCs and supporting cells in the organ of Corti and vestibular system (8). In the organ of Corti, tricellulin spans the entire unique depth of the tTJs between HCs and supporting cells (8,19), providing an opportunity to investigate mislocalization of tricellulin by light microscopy. Disruption of tricellulin along the length of tTJs in the organ of Corti was evaluated by image analysis in *Ildr1^w^* mice aged P2–P14, prior to OHC degeneration observed by SEM and myosin VIIa immunofluorescence staining (Figs 5B and 6D–E and Supplementary Material, Fig. S4). The depth of the tricellulin immunofluorescence signal was measured and compared with the depth of TJs demarcated by ZO-1 immunoreactivity as a ratio of distribution (ZO-1:tricellulin). From P2-5, tricellulin appeared to be normally localized in *Ildr1^w−/−^* mice (mean 0.98 ratio of distribution, 4.7% difference) compared with control animals (mean 1.03 ratio of distribution, Student's *t*-test, *P* > 0.05, Fig. 7A and E). However, at ∼P6–P10, tricellulin mislocalization was observed in *Ildr1^w−/−^* mice (mean 0.94 versus mean 1.07 ratio of distribution, 13.3% difference, Fig. 7B and F, Student's *t*-test, *P* < 0.005). By P11, tricellulin was noticeably absent from the top portion (relative to the apical surface of the HC) of tTJs in *Ildr1^w−/−^* mice (mean 0.84 versus mean 1.06 ratio of distribution, 22.7% difference) and was predominantly localized at a lower point near the base of the tTJs (Student's *t*-test, *P* < 0.0001, Fig. 7C and G). In control P14 *Ildr1^w+/+^* mice, localization of tricellulin (Fig. 7D) is indistinguishable from it's localization in *Ildr1^w+/−^* heterozygotes (Supplementary Material, Fig. S4A and D). In 1 month old *Ildr1^w−/−^* mice that lack OHCs, tricellulin is present at the newly formed tTJs between three supporting cells (Supplementary Material, Fig. S4G). Additionally, tricellulin localization in the marginal cells of stria vascularis and vestibular sensory epithelia showed no detectable differences between control and *Ildr1^w−/−^* mice (Supplementary Material, Figs S4B, C, E, F, H and I, and S5).

### Ultrastructure of tTJs is abnormal in *Ildr1^w−/−^* mouse inner ear

**Figure 8.**
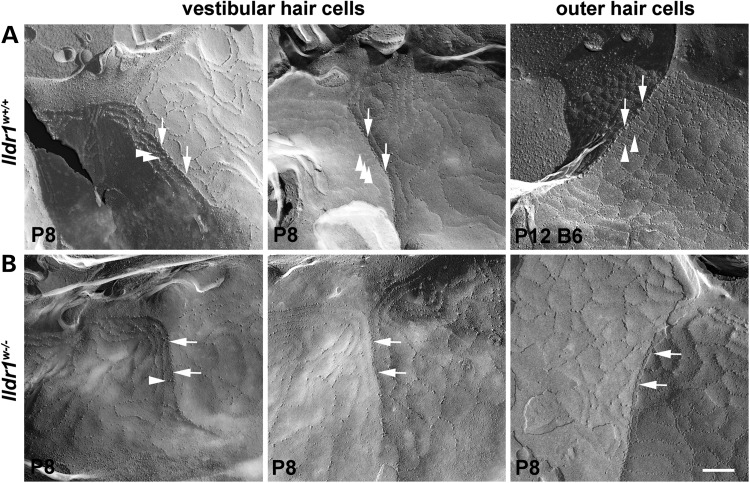
ILDR1 is required for the normal ultrastructure of the tTJ. Freeze-fracture images of the TJs in the tricellular region (tTJ) where a HC and two supporting cells are in contact. (**A**) P8 *Ildr1^w+/+^* mouse (left and central panels) and P12 C57Bl6/J wild-type mouse (right panel). (**B**) P8 *Ildr1^w−/−^* mouse. Vestibular sensory epithelium shown in the left and central panels (A and B), and OHC of the organ of Corti in the panels at the right. In the wild type (three panels in (A)), the contact between the three cells is defined as a prominent perpendicular strand of particles, which is the central element of the tTJ, to which several near horizontal strands, which are elements of bTJs, connect to tTJs. In the *Ildr1^w−/−^* animals (three panels in B), the central element of tTJs is less prominent with fewer particles (arrows) and the majority of the bTJ strands as they approach the tricellular junction run parallel to the tricellular junctional element rather than running perpendicular to it; only a few run into and join it (arrowheads). Scale bar: 200 nm.

The architecture of the outer leaflet of the plasma membrane can be examined by freeze-fracture electron microscopy (Fig. 8). In the wild-type mouse, freeze-fracture analyses of inner ear tTJs show strands running perpendicular to the OHC apical surface at the point where a HC and two supporting cells meet and where presumably tricellulin normally localizes. These strands appear as closely adjacent particles in a line. In the ILDR1-deficient mouse, the point of juncture between three cells is less defined and particles are not closely adjacent (Fig. 8B, arrows). Moreover, in the wild-type, some of strands that form the TAJ around the neck of the cell, run into and appear to join to the perpendicular strand at almost right angles. But in the mutant, there are almost no strands of the TJs that converge with a perpendicular strand. Rather, many of the strands defining the TAJ run parallel to the perpendicular line at the point of juncture of the three cells. These data indicate that ILDR1 may contribute to the ultrastructure of inner ear tTJs.

## DISCUSSION

### *Ildr1^w−/−^* and *Ildr1^k−/−^* mice recapitulate human deafness DFNB42

ILDR1 null mice (*Ildr1^w−/−^*) were generated to study the function of ILDR1 as a receptor in fat-stimulated CCK secretion due to its similarity with family member angulin-1/LSR, which mediates LDL uptake (29). We found that *Ildr1^w−/−^* and *Ildr1^k−/−^* young mice have profound hearing loss that recapitulates human deafness DFNB42 phenotype due to recessive mutations of *ILDR1* (16). In *Ildr1^w−/−^* mice, the absence of ILDR1 in cochlear tTJs results in a rapid degeneration of OHCs as early as P12, around the onset of hearing in mice (Fig. 5) (39,40), which coincides with the establishment of the mature EP in the basal turn of the cochlea. The presence of both normal appearing OHCs and IHCs in the organ of Corti and the generation of a normal negative EP in ILDR1 null mice prior to the onset of hearing indicate that the lack of functional ILDR1 in these mice does not affect development of the organ of Corti. Moreover, absence of ILDR1 does not alter recruitment of tricellulin to tTJs or localization of ZO-1 to bTJs of the postnatal inner ear during development before the onset of hearing. Pathological changes do begin to occur after P10, and there is an elevated ABR thresholds in *Ildr1^w−/−^* mice detected by the second postnatal week, The degeneration of OHCs and IHCs, and the eventual mislocalization of tricellulin, together with freeze-fracture analysis, demonstrate that ILDR1 is essential for the structural integrity and functionality of inner ear tTJs as well as for the long-term viability of HCs after the onset of hearing.

### Is human deafness due to mutations of *ILDR1* nonsyndromic or syndromic?

ILDR1 was shown to be a receptor that mediates the secretion of CCK (satiety hormone) in the presence of fatty acids or lipoproteins (29). This finding raises the possibility that ILDR1 could also have a receptor function in the inner ear for a presently unknown ligand in addition to serving as a TJ protein. Further experiments are needed to investigate this possibility. Although ILDR1 has a function in the small intestine, ILDR1 null mice are not outwardly or physiologically different from wild type. Neither *Ildr1^w −/−^* nor *Ildr1^k−/−^* mice appear to have a weight-gain phenotype associated with the loss of ILDR1 (Sanger institute mouse portal, www.sanger.ac.uk/mouseportal/) (29). To date there are no other discernable phenotypic abnormalities in these mice other than an auditory dysfunction described herein for both mutant alleles (*Ildr1^w−/−^* and *Ildr1^k−/−^*). However, original investigations of deaf DFNB42 individuals might have overlooked clinically relevant subtle abnormalities because the primary focus during ascertainment was limited to deafness (41). Our initial conclusion that mutations of human *ILDR1* cause exclusively nonsyndromic deafness DFNB42 may have to be revised as we learn more about the physiological consequence of a loss of ILDR1 in mouse (16).

### ILDR1 is not necessary for generation and establishment of an EP

Higashi and coauthors demonstrated that the angulin family members are important for maintenance of TJ barrier function in angulin-1/LSR knock-down EpH4 cells as detected by a significantly lower transepithelial electrical resistance (30,31). While these results indicated that a barrier function for ILDR1 in the inner ear was highly likely, the presence of a normal, positive EP provides evidence that loss of ILDR1 is not compromising the epithelial barrier in the cochlea to an extent that would diminish the EP. The generation of the EP depends on a low K^+^ concentration in the intrastrial fluid space, which is maintained by marginal cells of stria vascularis (13). The tTJs between marginal cells express both tricellulin and ILDR1 (8,30). The presence of a normal positive EP provides evidence that a paracellular leakage, if present, does not exceed the capacity of marginal cells to maintain the required low K^+^ concentration in the intrastrial space. For at least 2 months of age, the functional activity of the strial marginal cells, as well as the barrier function of the stria vascularis, remains intact during EP establishment in ILDR1-deficient mice. We also note that there is no obvious mislocalization of tricellulin in the marginal cell tTJs of the stria vascularis of *Ildr1^w−/−^* mice. However, we cannot exclude the possibility of partial tricellulin mislocalization from tTJs of marginal cells. It is also possible that in the stria vascularis, another angulin family member, such as LSR (previously reported to be expressed in the stria vascularis), ILDR2 (30,42), or an unidentified tTJ protein may fortify the marginal cell barrier and compensate for the loss of ILDR1 from these TJs.

### ILDR1 is necessary for retention but not recruitment of tricellulin at tTJs in the organ of Corti

Our data indicate that ILDR1 is not necessary for the recruitment of tricellulin to tTJs during development of the inner ear, unlike in angulin-1/LSR knock-down EpH4 cells (30). However, ILDR1 appears to be essential for maintaining the structure and integrity of the tTJs in the inner ear. Higashi and coauthors provided evidence for the recruitment of tricellulin by both FLAG-tagged ILDR1 and ILDR2 *in vitro*. Similarly, LSR-FLAG had previously been shown to recruit tricellulin using the same EpH4/LSR knock-down cells, and LSR is localized at tTJs of non-sensory epithelia in the inner ear (30,31). Notably, ectopic expression of some substitution mutations of ILDR1 disrupted ILDR1-driven tricellulin recruitment, indicating that there is likely an interaction between the two proteins. In this regard, our data indicates that ILDR1 is necessary for the retention of tricellulin at tTJs in the organ of Corti, but not for its recruitment. Mislocalization of tricellulin in *Ildr1^w−/−^* mice is detected prior to hair bundle disorganization observed at P12. It is possible that ILDR1 interacts with tricellulin to assist in maintaining the structure of the tTJs, but is not crucial for its initial recruitment to tTJs in the inner ear as shown for cells *in vitro* (30). However, we cannot exclude the possibility that ILDR1 may still be necessary for tricellulin recruitment in the other organs and tissues (30).

Another protein, which is absent in EpH4 cells, may be responsible for the proper localization of tricellulin in the inner ear of *Ildr1^w−/−^* mice. Generally, members of the angulin family do not have overlapping expression patterns and ILDR1 is the main family member expressed in the inner ear (30). Expression data from massively parallel signature sequencing libraries (http://www.ncbi.nlm.nih.gov/geo/query/acc.cgi?acc=GPL3835) of inner ear cDNAs indicate angulin-3/ILDR2 mRNA was not detected in the organ of Corti and vestibular sensory epithelia, but it is present in stria vascularis (42). Additionally, localization of angulin-1/LSR is different from that of ILDR1 in the inner ear (30). Functional compensation for the loss of ILDR1 in ILDR1 null mice does not occur since *Ildr1* homozygous mutant mice exhibit profound deafness suggesting a unique function for ILDR1 at tTJs. In a recent report (19), localization of ILDR1 was shown to be unaffected in tricellulin-deficient mutant mice, indicating that tricellulin does not recruit ILDR1 to the tTJs of the inner ear, similar to results obtained *in vitro* (30).

### Possible mechanisms of DFNB42 deafness and HC degeneration in *Ildr1^w−/−^* mice

The selective movement of ions, such as K^+^, across the reticular lamina of the cochlea, is related to the establishment of the negative EP (43–45). A lack of ILDR1, and subsequent dysfunction or degeneration of OHCs in *Ildr1^w−/−^* mice, presumably disrupts the movement of ions across the endolymph–perilymph barrier. OHC loss has been shown to impair the establishment of an anoxic EP (35). Therefore, OHC degeneration in ILDR1-deficient mice may account for the smaller amplitude of anoxic EP in these mice. However, loss of OHCs alone in ILDR1-deficient mice cannot explain the observed increase in ABR thresholds. OHC degeneration would lead to a loss of the cochlear amplifier and hence only a 40–60 dB increase in threshold (46). It seems likely that, due to loss of ILDR1, there is an additional dysfunction elsewhere in the auditory system.

Hair cell degeneration in *Ildr1* homozygous mutant mice may be due to malfunction of intracellular signaling, exposure of HCs to a toxic extracellular microenvironment, or both. The tricellulin knock-in mouse model of human DFNB49 deafness also showed an OHC degenerative phenotype in the presence of normal EP, similar to *Ildr1^w−/−^* mice (19). When mutant tricellulin mice were mated with POU3F4-deficient mice that do not establish an EP and have a reduced concentration of K^+^ as well as other ions in the endolymph (47), HCs in double mutants deficient both for tricellulin and POU3F4 remained intact. This indicated that HC degeneration in these mice may be due to extracellular factors, such as a toxic microenvironment on the lateral walls of HCs due to the altered permeability of tricellular barriers between hair and supporting cells in the absence of tricellulin. Future experiments will determine if HC degeneration in *Ildr1^w−/−^* and *Ildr1^k−/−^* mice is a cell non-autonomous effect associated with tTJ breakdown.

The observations in ILDR1 null mice that OHC were affected earlier than IHC and that OHC in the basal and middle turns were more affected than OHC in the apical turn is consistent with a common pattern of cochlear damage that has been found in association with aging, noise trauma and drug-induced ototoxicity (48–50). Underlying reasons for this pattern may include qualitative differences between IHC and OHC. The lateral plasma membrane of an IHC is closely opposed to supporting cells while OHCs are surrounded by perilymph allowing for prestin-driven contraction and elongation of cell bodies in response to auditory stimuli. We hypothesize that these rapid cyclical contractions of OHC bodies, when tTJs lack ILDR1 and are compromised, permit influx of ions and molecules from the potassium-rich endolymph into perilymph creating a toxic microenvironment that trigger irreversible damage to OHCs. When the tTJ barrier is compromised, increased K^+^ exposure to the basolateral surfaces of OHCs in the organ of Corti may lead to prolonged depolarization of OHCs due to inhibition of K^+^ efflux from HCs during repolarization, which induces HC death (51). Alternatively, loss of tricellulin from the most apical region of the tTJ in *Ildr1^w−/−^* mice begins approximately at the time the Ca^2+^ gradient between endolymph and perilymph is being established, while the formation of the Na^+^ and K^+^ gradients are nearly complete (52,53). An early sign of degeneration at P6-P10, evidenced by tricellulin mislocalization in tTJ, may result in a leaky paracellular pathway with detrimental consequences for OHCs.

The tTJs are responsible for the majority of molecular and macromolecular flux within the paracellular space (26). While it is possible that alterations of the tTJ sealing elements may increase ionic paracellular flux, there may also be an increased permeability to ATP across the scala media. Exposure to high levels of sound has been shown to increase ATP concentration in the endolymph (54). Increased ATP concentration and/or impaired molecular flux through the paracellular pathway may invoke a downstream mechanism leading to HC death. The ability of the boundaries of the scala media of *Ildr1^w−/−^* and *Ildr1^k−/−^* mice to maintain a positive EP suggests that the ionic paracellular permeability is largely functionally intact, and the selective increase of flux of other molecules from endolymph such as, for example, ATP could be a more likely explanation for OHC degeneration.

Vestibular HCs in *Ildr1^w−/−^* mice do not degenerate. The vestibular end organs do not possess the same electrochemical gradient that is found in the auditory periphery and the basolateral surfaces of vestibular HCs are not as exposed to the surrounding liquid as compared with OHCs. We note that ultrastructural analyses of freeze-fracture replicas from vestibular epithelium of *Ildr1^w−/−^* mice show structural abnormalities similar to those observed in the tricellulin-deficient mice. These data suggest that the alterations of a barrier function of the inner ear tTJs in *Ildr1^w−/−^* mice and the mechanism of OHC death may be similar to those observed in tricellulin-deficient mice.

The results of our study consolidate the importance of ILDR1 as a TJ protein necessary for HC survival, and for the integrity of the auditory system. Our data indicate that two tTJ proteins, tricellulin and ILDR1, are recruited to tTJs independently of one another. However, once in place, these two proteins may interact to maintain the structural integrity and function of tTJs, which are critical for normal hearing function in human and mouse.

## MATERIALS AND METHODS

### Mutant alleles of mouse *Ildr1*

All animals in this study were handled according to either the NIH Animal Care and Use Committee guidelines or the UK Home Office regulations and the UK Animals (Scientific Procedures) Act of 1986 (ASPA) under a UK Home Office license, and the study was approved by the King's College London Ethical Review Committee. Mice were culled using methods approved under these guidelines to minimize any possibility of suffering.

Two different mutant alleles of mouse *Ildr1* were used in this study. *Ildr1^Gt(D178D03)Wrst^*, abbreviated here as *Ildr1^w−/−^* knock-out mice, were generated on a Swiss Webster background in Rodger Liddle's laboratory at the Duke Neurotransgenics facility using gene-trapped ES cell clone D178D03 TBV-2 (29). The targeting vector rFlipROSAβgeo (Cre) was integrated in intron 2 of *Ildr1* (197 bp internal to the exon 2/intron 2 boundary). *Ildr1* failed to amplify in a reverse transcription polymerase chain reaction (RT-PCR) assay using RNA obtained from homozygous mutant mice. The second mutant allele of *Ildr1* used in this study, *Ildr1^tm1(KOMP)Wtsi^*, designated here as *Ildr1^k−/−^*, was generated and maintained on a C56BL/6N background as part of The Wellcome Trust Sanger Institute Mouse Genetics Projects (project ID: 31366) (55,56). The targeting vector was inserted such that exons 3–5 were deleted, supposedly inactivating the gene and inserting a LacZ cassette containing a strong splice acceptor. A high-throughput phenotyping screen identified a hearing deficit in the homozygous mutants at 14 weeks of age (56), but did not detect any other phenotypic deficits from the wide range of assays carried out (www.sanger.ac.uk/mouseportal). A breeding colony was established using heterozygote crosses to generate the necessary genotypes for experimental work.

### Genotyping

The *Ildr1^w^* mice were genotyped as previously described (29), with some modifications. Primers were combined together in a multiplex PCR consisting of one forward primer: *Ildr1* forward (5′CTGTCCTTGCTAGTCACAGTCC) located in exon 2, and two reverse primers: *Ildr1* reverse (5′GCTGACTTGAGGTCCCACAT) located in intron 2, and *lacZ* reverse (5′CAAGGCGATTAAGTTGGGTAACG) located inside the β-Geo cassette (Fig. 2B). Together, the two *Ildr1* primers yield a PCR 464 bp product using genomic DNA from both wild-type and heterozygote mice as a template. The *Ildr1* forward and *lacZ* reverse primers amplify a 1215 bp product in all *Ildr1^w+/−^* and *Ildr1^w−/−^* mice. Together, heterozygote genomic DNA samples yield both PCR products in the multiplex PCR while wild-type and homozygous mutant mice only result in their respective PCR products (Supplementary Material, Fig. S6A). *Ildr1^w^* mice continued to be bred using heterozygote crosses on their Swiss Webster background with no further back-crossing.

To genotype the *Ildr1^k^* allele, genomic DNA was extracted and used as the template for short-range PCR using the forward primer (5′TAATGCAGACAGGCTTTGGA), and the reverse primer (5′GCGAACTTCCCTCTGATTGT) for the wild-type allele to yield a 233 bp PCR product. The forward primer for the mutant allele was the same as that for the wild type, and the reverse primer was (5′TCGTGGTATCGTTATGCGCC) to yield a 159 bp PCR product. The presence or absence of the cassette (Fig. 2C) was confirmed by testing for the neomycin resistance sequence using the forward primer (5′CAAGATGGATTGCACGCAGGTTCTC) and the reverse primer (5′GACGAGATCCTCGCCGTCGGGCATGCGCGCC) to produce a 600–700 bp PCR product (Supplementary Material, Fig. S6B).

### ABR and DPOAE measurements

Auditory testing of *Ildr^w−/−^* and *Ildr1^k^* mice and their respective littermate controls was completed in different laboratories. *Ildr^w−/−^*, *Ildr1^w+/−^* and *Ildr1^w+/+^* littermates were evaluated at the Mouse Auditory Testing Core Facility, NIDCD. Mice were analyzed and tested in a serial, non-terminal fashion at three ages: 2, 4 and 8 weeks of age. Mice were anesthetized via an intraperitoneal (IP) injection of a solution of Ketamine (56 mg/kg) and DexDomitor (Pfizer) (0.375 mg/kg). Pre-weaned mice received the required dosage diluted 1:1 in a warm saline solution to have a reasonable volume to inject. All mice were tested inside a sound-proof booth (Acoustic Systems) while resting on a heating pad connected to a temperature controller and rectal probe to maintain body temperature near 37°C.

Both DPOAEs and ABRs were measured in the right ear only using Tucker-Davis Technologies (TDT; Alachua, FL, USA) hardware (RZ6 Multi I/O processor, MF-1 speakers) and software (BioSigRz, v. 5.1). DPOAEs were recorded first using two TDT MF-1 speakers and an ER-10B+ microphone (Etymotic, Elk Grove Village, IL) coupled to the mouse's ear using a modified pipette tip (a 10 µl pipette tip was trimmed such that length = 10 mm and the diameter at the tip opening = 2 mm). DPOAE (2f1 − f2) levels were obtained in response to two primary tones at f1 = 65 dB SPL and f2 = 55 dB SPL with f2 varied between 4–44.8 kHz (5 pts/octave) and f2/f1 = 1.25. The mean noise floor was calculated from three points sampled above and three points sampled below the 2f1 − f2 frequency. Output data presented in dB V were converted to dB SPL offline based on the ER-10B+ microphone's calibration voltage (dB SPL = 20*log([10^(dBV/20)^]/.05) + 93.9).

ABR thresholds were determined by presenting Blackman-gated tone-burst stimuli (3 ms duration, 29.9/s, alternating polarity) at four frequencies (8, 16, 32 and 40 kHz). Subdermal needle electrodes were placed at the vertex and beneath each pinna, with the non-test (left) ear serving as the ground. Stimuli were delivered via a TDT MF-1 speaker in a closed-field configuration (PVC tubing provided with the speaker terminating in a modified pipette tip of the same dimensions as the one used with the DPOAE microphone). Responses were amplified (20×), filtered (0.3–3 k Hz) and digitized (25 kHz). Thresholds were determined through visual inspection of stacked waveforms (average of 512–1024 artifact-free responses per waveform). The stimulus initially was presented at 80 dB peSPL and then decreased in 10 dB steps until the waveform disappeared. The stimulus was then increased and decreased in 5 dB steps until the lowest stimulus level that produced a repeatable waveform (ABR threshold) was determined. At least two waveforms (1024 responses each) were obtained for stimulus levels at and near the ABR threshold to ensure repeatability of the response. If no response was evident at 80 dB peSPL, the stimulus level was increased to 90 dB peSPL and testing proceeded as described above. When no repeatable waves were visible at the highest stimulus level tested (90 dB peSPL), the threshold was designated as 100 dB peSPL for subsequent analyses.

*Ildr1^k−/−^*, *Ildr1^k+/−^* and *Ildr1^k+/+^* mice aged 2, 4 and 8 weeks were evaluated using ABRs only as previously described at the Wolfson Centre for Age-Related Diseases, King's College London (57). Mice were anesthetised by IP injection of a solution of Ketamine (1 mg/g) and Xylazine (0.01 mg/g) and placed on a homeothermic heating blanket. Subdermal needle electrodes were inserted as indicated above. Responses were filtered (0.3–3 kHz) and ABR thresholds were determined for click stimuli (10 µs, 42.6/s, condensation polarity) and tone pips (5 ms duration, 42.6/s) at eight frequencies between 3 and 42 kHz. Stimuli were presented in the free-field via a loudspeaker at a distance of 20 cm at levels ranging from 0 to 95 dB SPL (custom software and TDT hardware (57)). When the response cannot be detected at 95 dB SPL, threshold was arbitrarily assigned to be 95 dB SPL, the maximum sound level tested at King's College London. Response thresholds for each stimulus were estimated from the resulting ABR waveforms and used to construct an audiometric profile for the mouse. Thresholds were determined by visual inspection of stacked waveforms by identifying the lowest stimulus level with a recognizable waveform (average of 256 sweeps per waveform).

### EP measurements

EP measurements for *Ildr1^w−/−^* mice and wild-type littermates were conducted in the Section of Molecular Biology and Genetics, NIDCD. Mice were anesthetized with tribromoethanol (Sigma-Aldrich) at a dose of 0.35 mg/g body weight per mouse. EP measurements were made using glass microelectrodes in adult (*n* = 7 normoxic, *n* = 5 *Ildr1^w+/+^*, 4 *Ildr1^w−/−^* anoxic) and P10 mice (*n* = 6 *Ildr1^w+/+^*, *n* = 7 *Ildr1^w−/−^*). Glass microelectrodes, consisted of tips of glass pipettes and silver/chloride electrodes, were prepared as described (58) using a P-97 Flaming/Brown micropipette puller (Sutter Instrument, Novato, CA, USA). Procedures were developed by modifying previously described protocols (53,59,60). Measurements were made in the basal turn of the cochlea by a round-window approach through the basilar membrane of the first turn. Anoxia was induced by intramuscular injection of succinylcholine chloride (0.1 µg/g body weight per mouse) after establishment of deep anesthesia followed by additional injection of tribromoethanol (*n* = 5, 4 for adult mice, *n* = 6, 7 for P10 mice). Data were recorded digitally (Digidata 1440A and AxoScope 10; Molecular Devices, Sunnyvale, CA, USA) and analyzed using Clampfit 10 (Molecular Devices).

EP measurements in *Ildr1^k^* mice were done at the Wolfson Centre for Age-Related Diseases, King's College London. Mice were anesthetised with an IP injection of urethane (0.1 ml/10 g of a 20% solution). Negative, anoxia EP, in addition to the normal positive EP, was measured in mice aged 2 weeks (P14, *n* = 4 *Ildr1^k−/−^*, *n* = 6 *Ildr1^k+/−^* and *n* = 4 *Ildr1^k+/+^*). Positive, normoxic EP was measured in mice aged 10 weeks (P67–P73, *n* = 7 *Ildr1^k−/−^*, *n* = 4 *Ildr1^k+/−^* and *n* = 4 *Ildr1^k+/+^*). A tracheal cannula was inserted into the left bulla to reveal the wall of the cochlea and a small hole was made over the basal turn of the cochlea. The tip of a 150 mm KCl-filled glass microelectrode was then inserted into the scala media. The EP of *Ildr1^k−/−^*, *Ildr1^k+/−^* and *Ildr1^k+/+^* littermates was measured as a stable positive potential, referenced to an Ag–AgCl pellet placed under the dorsal skin of the neck (34). Once the stable resting EP had been recorded, the electrode was left *in situ* and the mouse injected with an overdose of urethane. Following onset of anoxia, the potential fell rapidly and the maximally negative (anoxia) EP was measured (34).

### Immunocytochemistry and tricellulin mislocalization analyses

To evaluate morphological changes in the organ of Corti of *Ildr1^k−/−^* mice, after measurement of EP at P14, inner ears from homozygous and control littermates were fixed with 4% paraformaldehyde (PFA) for 2 h at room temperature (RT). After rinsing with phosphate buffered saline (PBS), the inner ears were decalcified with 10% EDTA for 4 h at RT and then dissected to expose the organ of Corti. In order to visualize the stereocilia bundles, the samples were stained with Rhodamine phalloidin (Life Technologies) diluted in 0.05%Triton-X/PBS for 40 min and after rinsing with PBS mounted in ProLong Gold antifade media (Life Technologies). Specimens were imaged using a Zeiss LSM 710 confocal microscope. Each organ of Corti was examined at 10% intervals along the length of the cochlear duct from the base.

To visualize possible HC degeneration and other morphological alterations in the organ of Corti of *Ildr1^w−/−^* mice, cochleae were extracted and fixed overnight at 4°C in 4% PFA diluted in 1× PBS at various ages. Organ of Corti and vestibular sensory epithelia were then microdissected in 1× PBS. The tectorial membrane was removed to expose the sensory epithelium. The stria vascularis with the underlying spiral ligament were detached from the organ of Corti. Following dissection, samples were permeabilized with 0.5% Triton X-100 for 20 min at RT, blocked at RT for 3 h in 2% protease-free bovine serum albumin (BSA), fraction V (Roche Diagnostics) containing 5% goat serum (Gibco, Life Technologies) diluted in 1 × PBS, filtered through a 0.22 µm filter (Millex-GP, Millipore). To visualize HC bodies, fixed samples were probed overnight at 4°C with a validated (37) commercial primary antibody against myosin VIIa (Proteus Biosciences).

To label TJ proteins, samples were fixed for 20 min on ice using fresh, cold 10% trichloroacetic acid (TCA, Ricca Chemical Company), followed by an immediate transfer into 1× PBS. Tissues were then dissected and permeabilized with 0.5% Triton X-100 for 20 min at RT. Blocking commenced for 2–3 h at RT using a 2% BSA diluted in a 1× PBS solution filtered with a 0.22 µm filter. Samples were dual-probed overnight at 4°C with a mouse monoclonal anti-ZO-1 antibody (Life Technologies) to label TJs and either rabbit polyclonal anti-tricellulin (Life Technologies) or rabbit polyclonal anti-ILDR1 (Sigma-Aldrich) antibodies.

Following primary antibody incubation, tissue samples were washed three times with 1× PBS. Samples were then probed with corresponding secondary antibodies diluted in blocking solution (Life Technologies) for 20 min at RT. To visualize F-actin in myosin VIIa stained samples, tissues were simultaneously stained with rhodamine-conjugated phalloidin (Life Technologies). Samples were washed three additional times with 1× PBS and mounted on slides using ProLong Gold antifade reagent (Life Technologies). Z-stack acquisitions of all immunostained samples were imaged using a LSM780 confocal microscope (Zeiss Microimaging Inc.) equipped with a 63×, 1.4 NA objective and ZEN2012 software.

Tricellulin mislocalization in *Ildr1^w−/−^* mice was analyzed using Volocity™ 3D Image Analysis Software (PerkinElmer Inc., Akron, OH, USA). All organ of Corti samples used for analysis were imaged at 1.5× zoom using a 63×, 1.4 NA oil objective. For analysis, background signal was set as threshold for individual images and objects in red (ZO-1) and green (tricellulin) cannels were automatically identified by the software. Centers of identified objects were designated as centroids and centroid distances measured from the top of sample tissues were obtained from both channels and averaged for comparison. Mislocalization was determined by a comparison ratio of ZO-1 depth-to-tricellulin depth (ratio of distribution) and the percent differences between control and experimental groups were calculated. Analysis included four to eight images from each prepared sample with images from all three cochlear turns.

### Freeze-fracture analysis of *Ildr1^w−/−^*mice

The inner ears of P8 *Ildr1^w+/+^* and *Ildr1^w−/−^* littermates were removed from animals after decapitation, and the cochleae were dissected out in Leibowitz media (L-15, Invitrogen). The round and oval windows at the base of the cochlea were cleaned, membranes removed and a small hole was made in the apex of cochlear capsule. Cochleae were gently perfused with 2.5% glutaraldehyde in 0.1 m cacodylate buffer with 3 mm CaCl_2_ through these openings, followed by immersion in the same fixative overnight at 4°C. The sensory epithelia of the inner ear were microdissected and incubated in 25% glycerol for at least 45 min prior to mounting on support planchettes using a yeast in glycerol paste as adhesive, and freezing in a rapidly stirred mixture of propane/isopentane (4:1) chilled in liquid nitrogen. Freeze-fracture replicas were obtained by routine procedures in a Balzers BAF400D apparatus (61). Replicas were viewed and imaged on a JEOL 1200EXII microscope. Digital images are adjusted for optimal contrast and brightness using Photoshop GS5 (Adobe) and are presented in reverse contrast.

### SEM

SEM examination and sample preparation were performed as described previously (62) with slight modifications. Briefly, *Ildr1w^+/+^* and *Ildr1w^−/−^* mouse cochleae were dissected out of the temporal bones, fixed in 2.5% glutaraldehyde in 0.1 m cacodylate buffer (EMS) supplemented with 2 mm CaCl_2_ for 2 h at RT and dehydrated in graded series of ethanol (EtOH). Specimens were then transferred from 100% EtOH to 100% acetone, placed into metal mesh baskets (Ted Pella Inc., Redding, CA, USA), critical point dried from liquid CO_2_ (CPD030 Critical point Dryer, BAL-TEC AG, Florida, USA), and finally sputter-coated by 4–5 nm thick platinum using turbo-pumped sputter coater Q150T (Quorum Technologies, UK). Samples were mounted on aluminum studs (Electron Microscopy Sciences, Hatfield, PA, USA) and imaged using a field emission scanning electron microscope (S-4800, Hitachi, Japan).

### Statistics

Statistical analysis of EP data for *Ildr1^w−/−^* mice was performed using a two-tailed distribution Student's *t*-test of independent variables and graphed using Excel. For each data set on tricellulin mislocalization, the D'Agostino and Pearson omnibus normality test in GraphPad Prism 5 software (La Jolla, CA) was performed to determine Gaussian distribution. Statistical differences between data were then determined by a two-tailed distribution Student's *t*-test of independent variables. EP analysis for *Ildr1^k^* mice was performed by ANOVA. A resulting *P* value of <0.05 was considered to be significant for all statistical tests represented by asterisks in graphs. All graphically represented data are shown as an average ± SD.

## SUPPLEMENTARY MATERIAL

Supplementary Material is available at *HMG* online.

## FUNDING

This work was supported in part by the Wellcome Trust (WT100669MA to K.P.S.), the Medical Research Council (to K.P.S.) and the Rosetrees Trust (A.F.). This work was supported by National Institutes of Health (DK098796 and DK091946 to R.A.L., NIH-R01-DC012151 to P.W.) and the National Institute on Deafness and Other Communication Disorders—Intramural Research Program (DC000060-14 to A.N. and DC000039-18 to T.B.F.). Funding to pay the Open Access publication charges for this article was provided by the NIDCD/NIH from T.B.F.'s budget.

## Supplementary Material

Supplementary Data
