# Supplementary material for: ILDR1 null mice, a model of human deafness DFNB42, show structural aberrations of tricellular tight junctions and degeneration of auditory hair cells
Source: Hum Mol Genet. 2014 Sep 12;24(3):609–24. doi: 10.1093/hmg/ddu474 (PMC4291242; doi:10.1093/hmg/ddu474)
Supplement: Supplementary Data [file supp_24_3_609__index.html]

ILDR1 null mice, a model of human deafness DFNB42, show structural aberrations of tricellular tight junctions and degeneration of auditory hair cells — ILDR1 null mice, a model of human deafness DFNB42, show structural aberrations of tricellular tight junctions and degeneration of auditory hair cells — Supplementary Data 

# ILDR1 null mice, a model of human deafness DFNB42, show structural aberrations of tricellular tight junctions and degeneration of auditory hair cells

## Supplementary Data

Supplementary Data

**Files in this Data Supplement:**

- Supplementary Data - Pdf file
