# Supplementary material for: ILDR1 null mice, a model of human deafness DFNB42, show structural aberrations of tricellular tight junctions and degeneration of auditory hair cells
Source: Hum Mol Genet. 2014 Sep 12;24(3):609–24. doi: 10.1093/hmg/ddu474 (PMC4291242; doi:10.1093/hmg/ddu474)
Supplement: Supplementary Data [file supp_ddu474_ddu474supp.pdf]

# Supplemental Figures and Legends

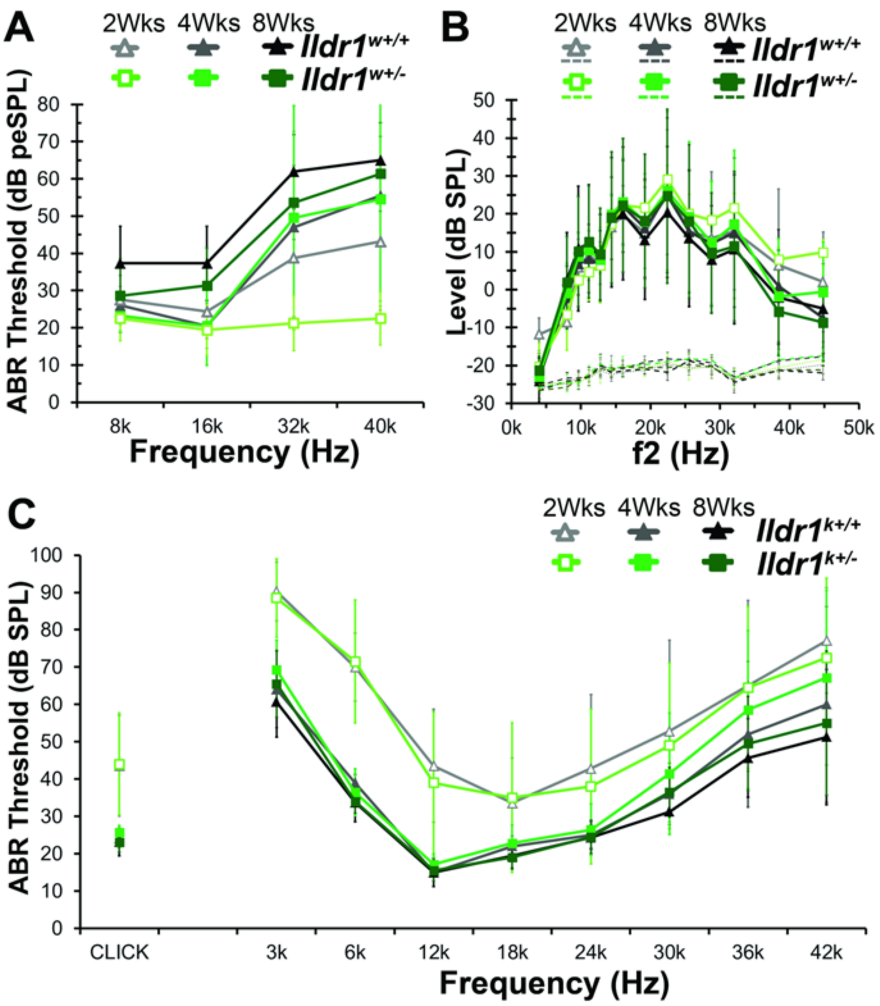


**Figure S1.** ABR and DPOAE data for phenotypically wild type heterozygotes. (**A**) ABR thresholds of *Ildr1*$^{w+/-}$ littermates are comparable to *Ildr1*$^{w+/+}$ controls. High frequency hearing loss at 4 and 8 weeks of age is observed for both *Ildr1*$^{w+/-}$ and *Ildr1*$^{w+/+}$ mice (n=8, 11, 11 for 2, 4, and 8 weeks respectively, per genotype). DPOAE levels (**B**) at 2, 4, and 8 weeks of age for *Ildr1*$^{w+/+}$ and *Ildr1*$^{w+/-}$ littermates. *Ildr1*$^{w+/+}$ and *Ildr1*$^{w+/-}$ mice have similar thresholds at all ages tested. Dotted lines represent noise floor corresponding to each age and genotype. (**C**) *Ildr1*$^{k+/-}$ mice (n=10, 7, 10 for 2, 4, and 8 weeks respectively) have normal thresholds compared to

*Ildr1*$^{k+/+}$ controls (n=7, 5, 8 for 2, 4, and 8 weeks respectively). Error bars (**A-D**) are shown as ± SD.

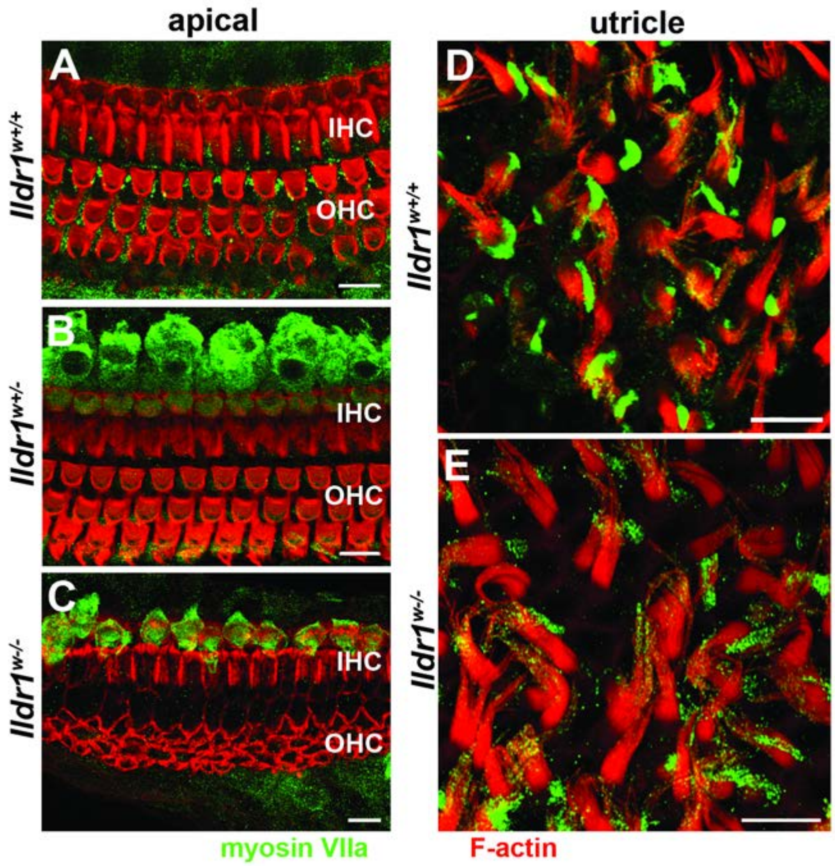


**Figure S2.** *Ildr1*$^{w-/-}$ vestibular hair cells and *Ildr1*$^{w+/-}$ auditory hair cells do not degenerate. Maximum intensity projections of the apical turn organ of Corti whole mounts for 1 month old (**A**) *Ildr1*$^{w+/+}$, (**B**) *Ildr1*$^{w+/-}$, and (**C**) *Ildr1*$^{w-/-}$ mice are shown. (**A**) *Ildr1*$^{w+/-}$ mice show no signs of hair cell degeneration. (**C**) *Ildr1*$^{-/-}$ mice show complete loss of OHCs by 1 month of age. Maximum intensity projections of utricle whole mounts from adult, 2 month old (**D**) *Ildr1*$^{w+/+}$ and (**E**) *Ildr1*$^{w-/-}$ mice show no sign of vestibular hair cell degeneration. Samples were stained for the hair cell body marker myosin VIIa (green) and probed with phalloidin to label F-actin (red). Scale bars: 10 μm.

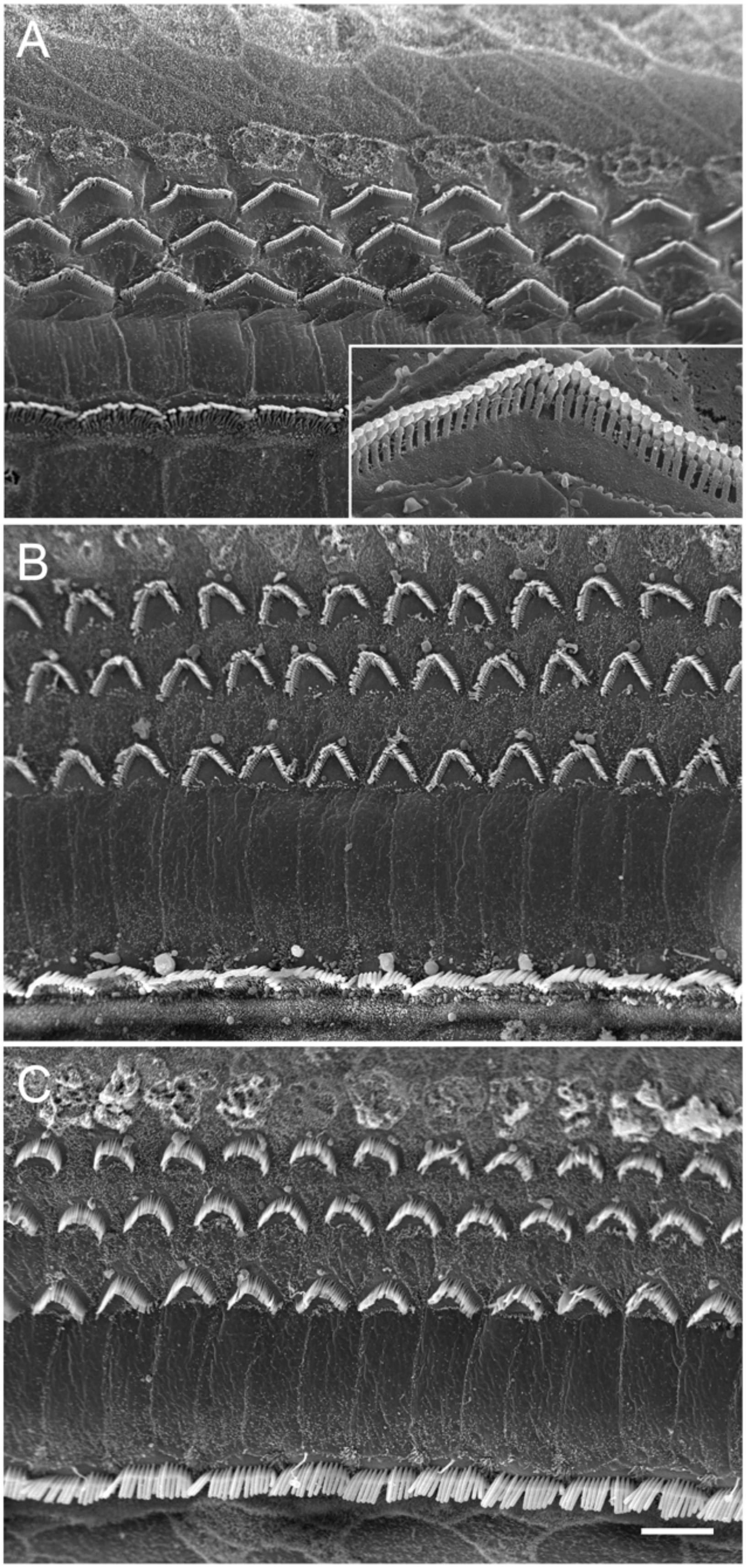
A
B
C

**Figure S3.** SEM images of P11 *Ildr1*$^{w-/-}$ organ of Corti. (**A**) basal, (**B**) middle, and (**C**) apical turns of the organ of Corti of a P11 *Ildr1*$^{w-/-}$ mouse show apparently normal surface morphology of IHC, OHC and supporting cells. Insert in (**A**) shows an enlarged view of a stereocilia bundle from an OHC located in the basal. Note that the tips of stereocilia in the shorter row are connected by tip-links to stereocilia of the adjacent longer row. Scale bar in C is 5 µm and applies to A and B.

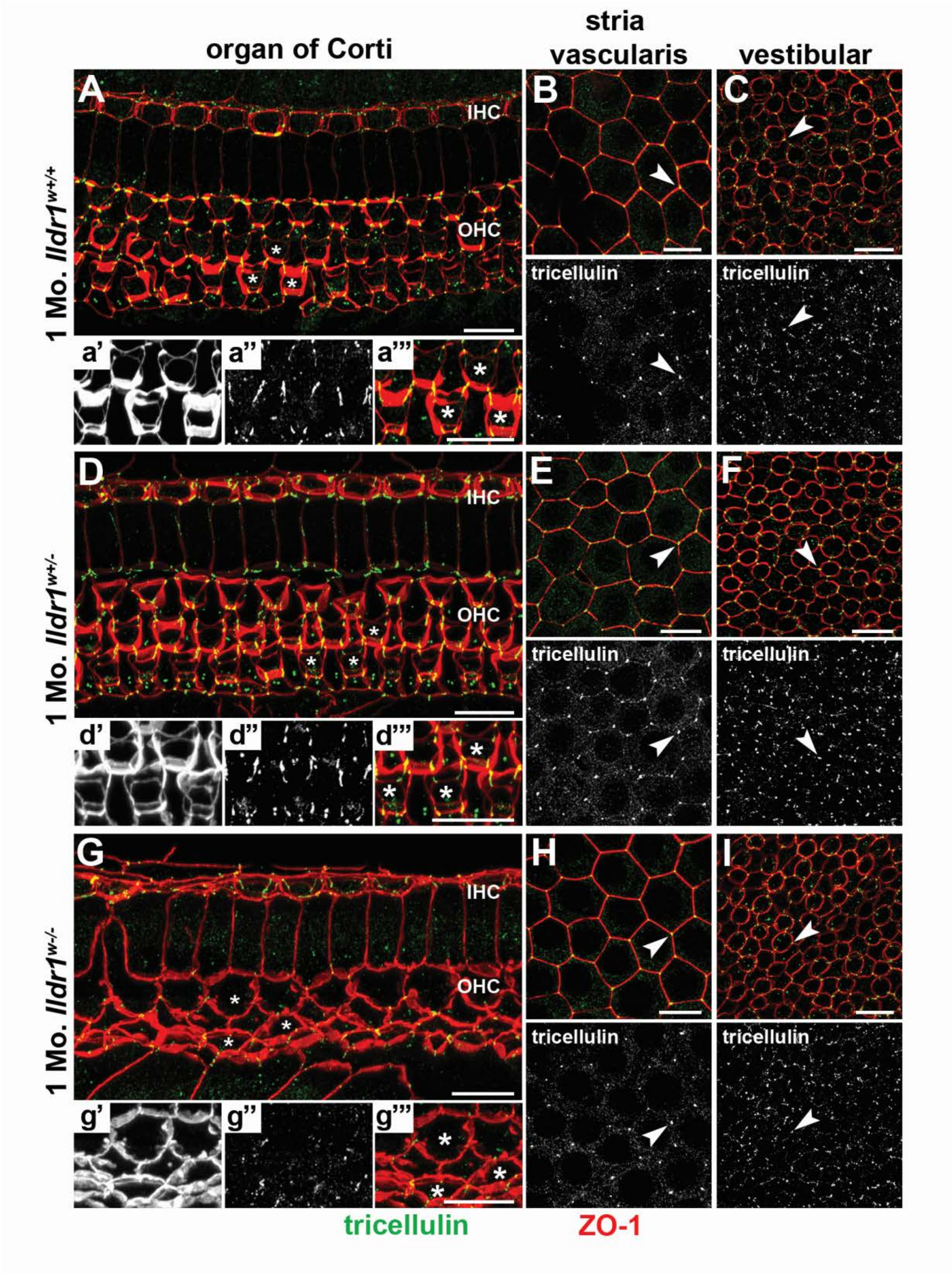


**Figure S4.** Tricellulin is present in 1 month old *Ildr1*$^{-/-}$ mouse organ of Corti that lack OHCs. (**A-I**) 3D reconstructions of z-stack confocal images from whole-mount organ of Corti samples, probed with antibodies directed against tricelluin (green) and ZO-1 (red). All panels in grey scale

show the green channel (tricellulin) (**A, a’-a’’’**) Tricellulin is localized along the entire tTJ depth in 1 month old *Ildr1*$^{w+/+}$ mice in the organ of Corti and also localized to tTJs in (**B**) stria vascularis and (**C**) vestibular (ampule) tissues. (**D, d’-d’’’**) Tricellulin is also localized along the entire tTJ depth in 1 month old *Ildr1*$^{w+/-}$ heterozygous mice in the organ of Corti and in tTJs in (**E**) marginal cells of stria vascularis and in (**F**) sensory epithelium of saccule. (**G, g’-g’’’**) Tricellulin is still detected in the newly formed supporting cell tTJs in 1 month old *Ildr1*$^{w-/-}$ mouse organ of Corti that lack OHC and is present at tTJs of both (**H**) marginal cells of stria vascularis and (**I**) vestibular sensory epithelium of saccule. Arrowheads in **B-C**, **E-F** and **H-I** point to tTJ and corresponding tricellulin signal. Asterisks in **A, a’’’, D, d’’’, G, g’’’** indicate hair cells depicted in magnified panels. Scale bars: 10 µm.

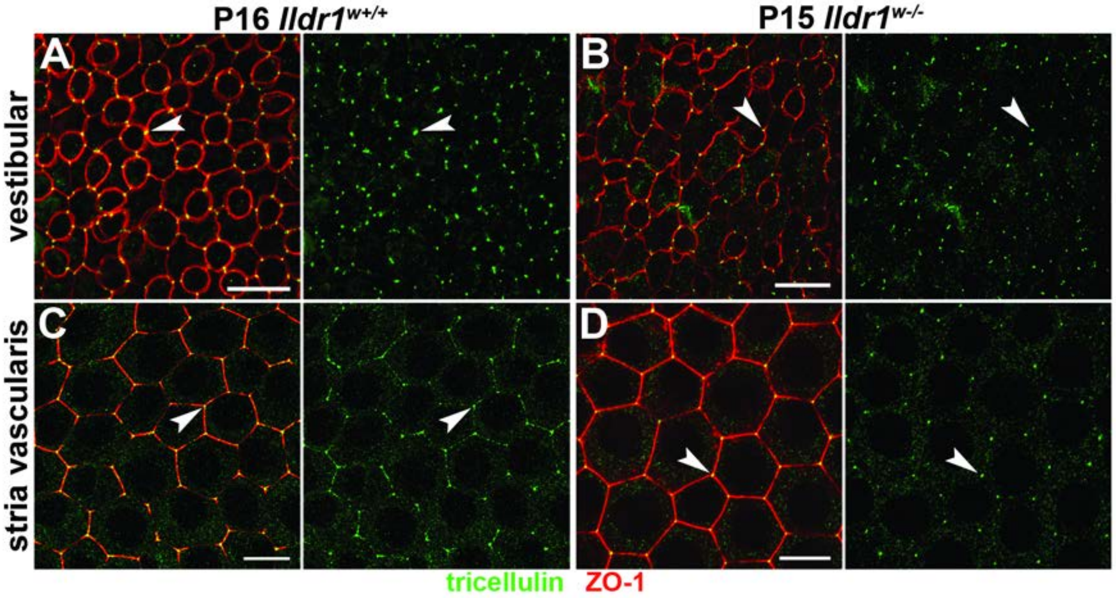


**Figure S5.** Tricellulin localizes normally to tTJs of vestibular sensory epithelia and stria vascularis marginal cells in *Ildr1*$^{w-/-}$ mice. (**A**) Saccule of P16 *Ildr1*$^{w+/+}$ mouse and (**B**) ampule from P15 *Ildr1*$^{w-/-}$ mouse show tricellulin localization at tricellular contacts of vestibular sensory epithelia.Normal localization of tricellulin (green) is seen at tricellular contacts of stria vascularis

marginal cells in (**C**) P16 *Ildr1*$^{w+/+}$ and (**D**) P15 *Ildr1*$^{w-/-}$ mouse tissues. Arrowheads point to tricellular contacts and corresponding tricellulin signal in split channel images. Samples were counterstained with an antibody to ZO-1 (red). Scale bars: 10 µm.

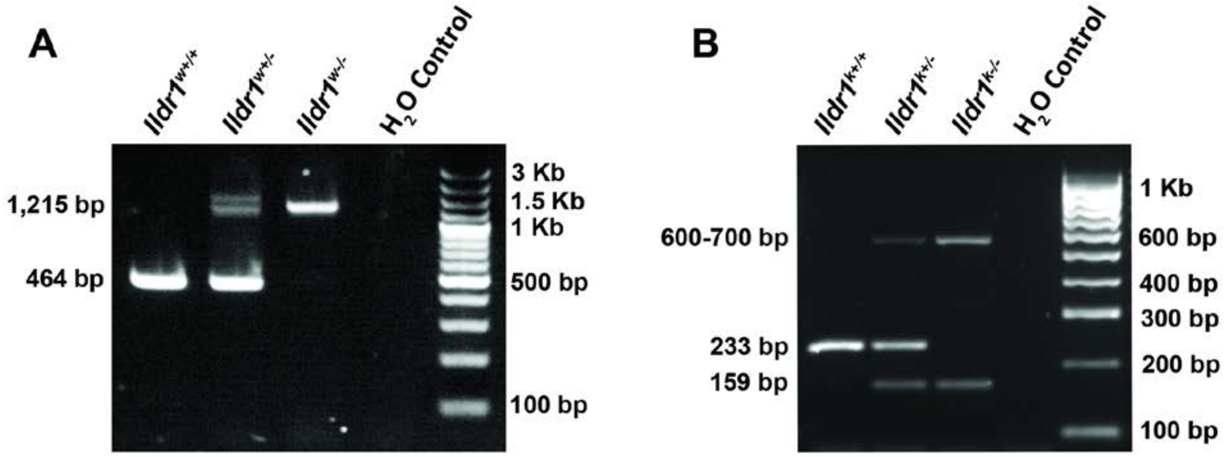


**Figure S6.** *Ildr1*$^{w-/-}$ and *Ildr*$^{k-/-}$ genotyping results. (**A**) Multiplex PCR showing product sizes for *Ildr1*$^{w+/+}$ (464 bp), *Ildr1*$^{w+/-}$ (464 bp, 1,215 bp), and *Ildr1*$^{w-/-}$ (1,215 bp) mice. Double band at 1,215 bp in heterozygous *Ildr1*$^{w+/-}$ mice is likely a heteroduplex and is not seen in *Ildr1*$^{w+/+}$ mice, *Ildr1*$^{w-/-}$mice, or when only amplified with the primer set for the mutant allele. (**B**) Multiplex PCR showing product sizes for *Ildr1*$^{k+/+}$ (233 bp), *Ildr1*$^{k+/-}$ (233 bp, 159 bp) and *Ildr*$^{k-/-}$ (159 bp) mice. Product band from Neo (600-700 bp) confirms the presence or absence of the cassette.
